# Polyelectrolyte Multilayer: Caged PTFE Particles in Water for Continuous, Sonochemical H_2_O_2_ Synthesis

**DOI:** 10.1002/advs.77281

**Published:** 2026-08-24

**Authors:** Rong Wang, Bo Wang, Tengyue Fu, Yue Yang, Chongling Cheng, Zhenhui Qi, Dayang Wang

**Affiliations:** ^1^ State Key Laboratory of Digital Medical Engineering School of Biological Science and Medical Engineering Southeast University Nanjing People's Republic of China; ^2^ State Key Laboratory For Inorganic Synthesis and Preparative Chemistry College of Chemistry Jilin University Changchun People's Republic of China

**Keywords:** hydrogen peroxide, hydrophobic interfaces, polyelectrolyte multilayers, PTFE particles, Sonochemistry, yolk–shell particles

## Abstract

The use of polytetrafluoroethylene (PTFE) particles to promote sonochemical H_2_O_2_ production has attracted growing interest, as cavitation bubbles formed at or near their perfluorinated surfaces provide favorable interfacial environments for generating reactive radicals from water and oxygen. However, their practical application is limited by poor dispersibility in aqueous media. Here, we report a yolk–shell particle (YSP) design, where PTFE cores are encapsulated within polyelectrolyte multilayer (PEM) shells assembled via layer‐by‐layer deposition of polyallylamine hydrochloride and poly(styrene sulfonate). This structure enables stable dispersion in water while maintaining exposure of hydrophobic PTFE surfaces through internal void spaces, thereby maximizing the ultrasound‐driven interfacial H_2_O_2_ generation efficiency of PTFE surfaces. As a result, continuous sonochemical conversion of H_2_O and O_2_ to H_2_O_2_ is achieved, with a maximum concentration of 2.5 mmol·L^−1^ over 12 h and a production rate of 3.34 mmol·g_cat_^−1^·h^−1^. Moreover, the YSP platform allows systematic evaluation of particle size and surface chemistry effects. This strategy not only enables the reuse of polymer waste as functional promoters but also facilitates the development of accessible, cost‐effective sonochemical H_2_O_2_ generators for applications in water purification, food safety, and healthcare.

## Graphical abstract

A yolk–shell PTFE‐based composite (YSP) enables tunable, robust hydrophobic–water interfaces for efficient, on‐demand sonocatalytic H_2_O_2_ generation and pollutant degradation.

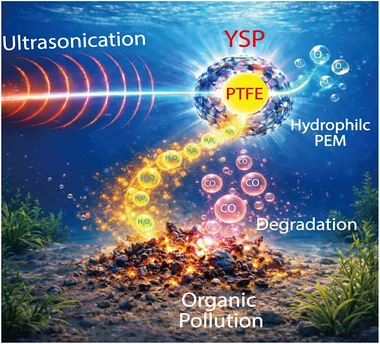

## 1 Introduction

Hydrogen peroxide (H_2_O_2_) is a versatile and environmentally benign oxidant widely used in chemical processing, environmental remediation, disinfection, and personal and public hygiene [1, 2]. Industrial H_2_O_2_ production is predominantly based on the anthraquinone process, which enables centralized manufacturing of high‐concentration H_2_O_2_. However, the energy‐intensive operation and inherent challenges associated with the storage, transportation, and handling of the concentrated H_2_O_2_ limit its deployment in decentralized and small‐scale applications, particularly in household and clinical settings [3, 4]. Therefore, developing safe and sustainable strategies for on‐demand generation of dilute H_2_O_2_ at the point of use is highly desirable.

To facilitate decentralized and on‐demand applications, a dazzling variety of portable approaches have been developed to convert H_2_O and O_2_ into H_2_O_2_, generally relying on electrocatalytic, enzymatic, photocatalytic, and plasma‐assisted catalytic processes, often with the aid of sacrificial agents or additional chemical inputs [5, 6, 7, 8, 9]. In this context, it is of burgeoning interest to harness microdroplets and microbubbles for catalyst‐free H_2_O_2_ synthesis, since large electric field gradients across air/water interfaces – the underlying mechanism still remains under debate – can readily decompose H_2_O to highly reactive hydrogen and hydroxyl radicals in the interfacial region [10, 11]. Hydroxyl radicals (HO^•^) and superoxide radical anions (O_2_^•−^) can subsequently participate in H_2_O_2_ formation [12, 13, 14, 15]. However, pristine PTFE particles exhibit extremely poor dispersibility and strong aggregation in water, which significantly limits the accessible PTFE–water interfacial area and hinders their practical application in continuous and scalable sonochemical H_2_O_2_ production [16].

Here, we address this limitation by constructing yolk–shell particles (YSPs), in which hydrophobic PTFE particles, as well as other hydrophobic polymer particles, are encapsulated as movable yolks within hydrophilic polyelectrolyte multilayer shells. The particles are prepared through layer‐by‐layer (LbL) assembly of oppositely charged polyelectrolytes, followed by pH‐induced separation of the hydrophilic shell from the hydrophobic core. The hydrophilic shell enables stable aqueous dispersion, whereas the internal void preserves the accessibility of the perfluorinated PTFE surface and provides a confined interfacial region favorable for gas‐nucleus retention and heterogeneous cavitation nucleation. The resulting yolk–shell particles enable continuous ultrasound‐driven conversion of H_2_O and O_2_ into H_2_O_2_ at a production rate of 3.34 mmol g_cat_^−1^ h^−1^. This platform further allows the effects of particle size and surface chemistry on H_2_O_2_ production to be quantitatively evaluated and supports the development of a portable ultrasonic H_2_O_2_ generator capable of removing more than 99.9% of pesticide residues from fruits and vegetables within several minutes [17, 18, 19].

## 2 Results and Discussion

### 2.1 Fabrication of Yolk–Shell Particles Enabling Excellent Water Dispersibility of PTFE Cores

The technical dilemma to employ PTFE particles as micro‐sized hydrophobic platforms to create cavitating bubbles with ultrasound is to effectively and stably disperse these highly hydrophobic particles into water without chemically diminishing their surface hydrophobicity for acoustic cavitation; let alone the high stability of perfluorinated surfaces against chemical alteration. Taking advantage of their notable propensity for hydrophobic interactions‐induced fouling onto hydrophobic surfaces, particularly in the presence of salt in water, here polyallylamine hydrochloride (PAH) and polystyrene sulfonate (PSS) were used for LbL assembly onto commercial PTFE particles with average sizes of 0.1, 0.2, 1, 5, 10, and 30 µm, respectively, in degassed water at the presence of 1.0 M NaCl (Figure S1). Compared with conventional LbL assembly on hydrophilic particles, degassing of water was essential for minimizing the hydrophobic interactions between PTFE particles and, in turn, improving their dispersibility in aqueous saline media, and the NaCl concentration was adjusted to 1.0 M to enhance hydrophobic interactions of polyelectrolyte chains with the PTFE particles in degassed aqueous media [20, 21].

**FIGURE 1:**
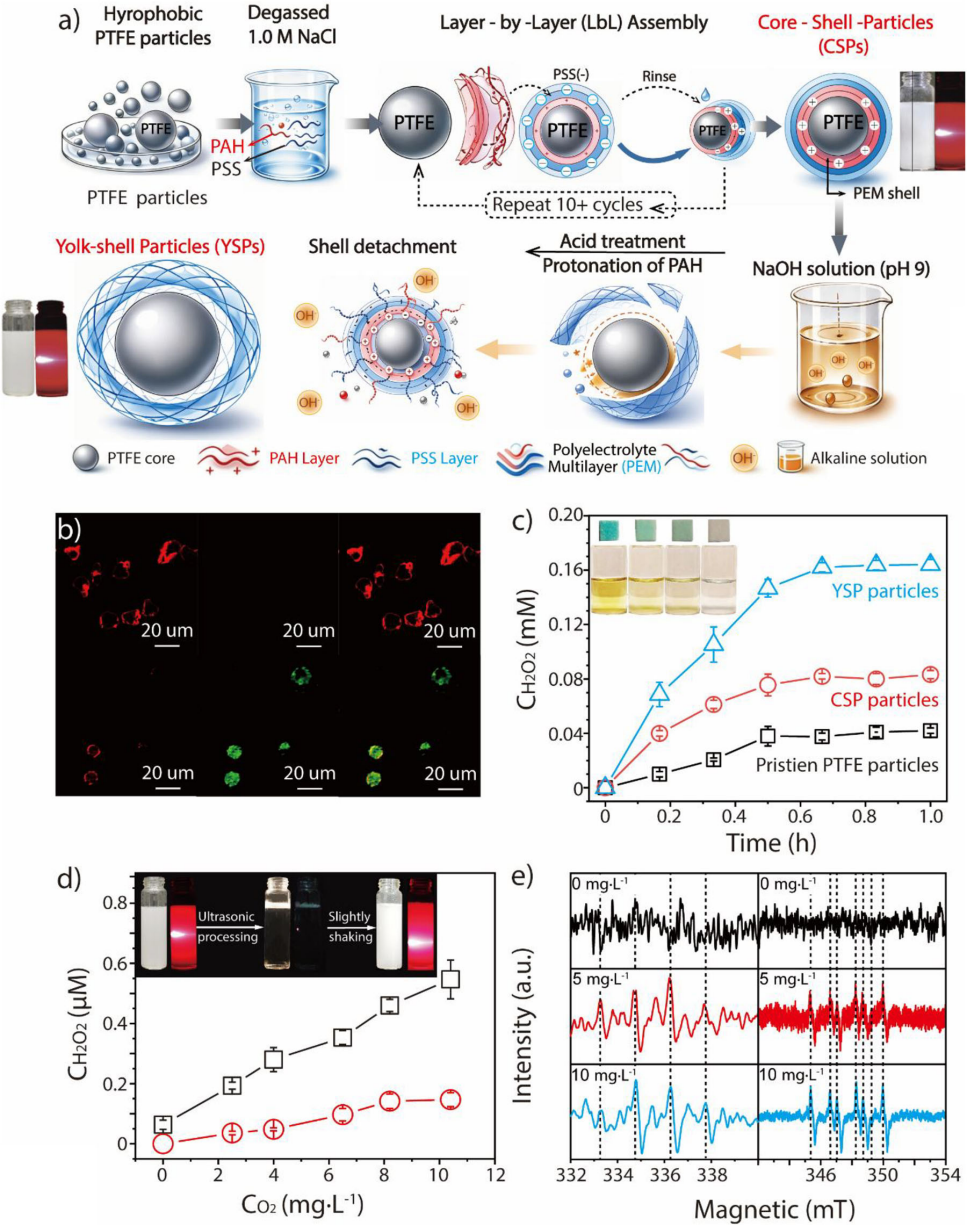
Construction of YSPs for sonochemical H_2_O_2_ generation. (a) Schematic illustration of fabrication of CSPs and YSPs via LbL growth of PAH/PSS multilayers on PTFE particles in water and utilization of their PTFE surfaces as a catalyst‐free platform to facilitate conversion of H_2_O and O_2_ into H_2_O_2_ under ultrasonic irradiation. The photos of CSPs (upper panel) and YSPs (lower panel) placed on water, followed by hand shaking to facilitate particle dispersion into water, are shown in the inset. (b) Confocal fluorescence images of CSPs (upper panels) and YSPs (lower panels), obtained after successive incubation in aqueous solutions of fluorescein sodium and Rhodamine 6G (R6G) for 12 h. Green fluorescence (fluorescein sodium), red fluorescence (R6G), and their merged overlay images are displayed to map the spatially selective adsorption of the dyes onto the particles. Note that the surfaces of PEM shells made of 10 PAH/PSS bilayers are negatively charged. The core–shell structure of CSPs permits only the electrostatic adsorption of positively charged R6G molecules onto the outer shell. In contrast, the YSPs architecture exposes the inner hydrophobic PTFE core, allowing it to adsorb both negatively charged fluorescein and positively charged R6G molecules via hydrophobic interactions, thereby confirming the spatial disengagement of the shell and the core. (c, d) Plots of $C_{H_{2}O_{2}}$ in water in the presence of pristine PTFE particles (squares), CSPs (circles), and YSPs (triangles) under ultrasonic irradiation (60 kHz, 100 W) versus irradiation time (c) and the O_2_ concentration ($C_{O_{2}}$) in water (d). The insets in Figure c show the photos of H_2_O_2_ test paper (upper) and glass vials of 10 mL of aqueous solutions of KI (0.1 M) in the absence and presence of PTFE particles (floating on the solution surface), CSPs and YSPs (right to left), obtained after 10 min of ultrasonication. The yellow color of the KI solutions and the bluish color of H_2_O_2_ test papers indicate the formation of H_2_O_2_ in water. The inset in Figure d shows the photos of an aqueous dispersion of YSPs (left panels), in which the particles are driven to float on the water surface after 1 h ultrasonication (middle panels) and then redispersed back in water by gentle shaking (right panel), visualized by the path of a light beam (Tyndall effect). (e) Electron paramagnetic resonance (EPR) spectra of spin‐trapped HO^•^ radicals (left panels) and O_2_^•−^ (right panels) generated in aqueous dispersions of YSPs after 30 min of ultrasonication at dissolved‐oxygen concentrations of 0 (black curves), 5 (red curves) and 10 (blue curves) mg L^−1^. The 0 mg L^−1^ condition corresponds to deaerated water (black curves). The dissolved‐oxygen concentrations were subsequently adjusted by bubbling O_2_/Ar gas mixtures of different compositions into the deaerated water. The dashed vertical lines indicate the characteristic hyperfine‐splitting positions of the corresponding radical adducts. The YSPs and CSPs used in Figure b–e contained PTFE yolks with an average size of 1 µm, and the particle concentration in water is 1 mg mL^−1^ [15].

The reasons for selecting PAH as a pH‐sensitive polycation with pKa of 8.7 were twofold: (1) it becomes partially positively charged at pH around its pKa, thus enabling electrostatic co‐assembly with pH‐independent, negatively charged PSS and enhancing hydrophobic interaction‐induced adsorption to the PTFE particles; (2) it is completely positively charged at pH below its pKa, thus enhancing electrostatic conjugation with PSS within the polyelectrolyte multilayer (PEM) shells and, meanwhile, promoting detachment of PEM shells from the PTFE particles. After alternating deposition of PAH and PSS was repeated for 10 cycles in degassed 1 M NaCl at pH 9 (Figure 1a and Figure S2,S3), the PTFE particles were uniformly and completely wrapped with PEM shells, and the resulting core–shell particles (CSPs) were incubated in 0.1 M HCl to ensure complete protonation of PAH chains to disengage the PEM shells from the PTFE cores.

In contrast to their CSP precursors, the resulting yolk–shell particles (YSPs) consist of movable PTFE yolks confined within hydrophilic polyelectrolyte multilayer (PEM) shells. The internal void allows water to access the perfluorinated PTFE surface while maintaining the structural separation between the PTFE yolk and the PEM shell (Figure 1a). To provide spatial evidence for this unique architecture, we designed a fluorescence‐dye labeling experiment based on the distinct surface properties of the PTFE yolk and the polyelectrolyte shell (Figure 1b). Specifically, the negatively charged hydrophilic PEM shell selectively interacted with cationic Rhodamine 6G (R6G) through electrostatic attraction, while excluding anionic fluorescein due to electrostatic repulsion. In contrast, the hydrophobic PTFE yolk adsorbed both R6G and fluorescein sodium, with adsorption mainly governed by hydrophobic interactions rather than electrostatic interactions.

Confocal fluorescence imaging revealed distinct spatial fluorescence distributions within YSPs. The merged fluorescence image showed a yellow fluorescence domain associated with the PTFE yolk due to the coexistence of red R6G and green fluorescein sodium signals, whereas the surrounding PEM shell remained predominantly red because of its selective adsorption of R6G. This spatially separated red‐shell/yellow‐yolk fluorescence pattern provides direct evidence for the coexistence and separation of the PEM shell and accessible PTFE yolk. In comparison, CSPs exhibited fluorescence mainly associated with the outer PEM layer and retained the charge‐selective adsorption behavior of the polyelectrolyte surface (Figure 1b). These distinct fluorescence distributions confirm the different interfacial properties of the PTFE yolk and PEM shell and support the formation of the yolk–shell architecture. Having established the structural characteristics of YSPs, we next evaluated their performance in ultrasound‐driven H_2_O_2_ generation.

### 2.2 Enhanced Sonochemical H_2_O_2_ Generation by YSPs

In stark contrast to pristine PTFE particles known for low surface energy and high surface hydrophobicity, as‐prepared CSPs and YSPs exhibited excellent dispersibility in water (Figure 1a and Figure S3), substantially improving their applicability in aqueous media. As a proof of concept, these particles were employed to facilitate the sonochemical conversion of H_2_O and O_2_ into H_2_O_2_. After dispersion in water and ultrasonication for 10 min under ambient conditions, pristine PTFE particles, CSPs and YSPs all promoted detectable H_2_O_2_ formation, whereas no measurable H_2_O_2_ was produced in the particle‐free control (Figure 1c). These results confirm the essential role of PTFE‐water interfaces in promoting sonochemical H_2_O_2_ generation. Previous studies have shown that acoustic cavitation alone can produce trace amounts of H_2_O_2_ through water sonolysis, while fluorinated polymer interfaces can further enhance H_2_O_2_ formation through interfacial processes [22].

Quantitative analysis of H_2_O_2_ concentration ($C_{H_{2}O_{2}}$) revealed that YSPs generated approximately twice as much H_2_O_2_ as CSPs and four times as much as pristine PTFE particles (Figure S4). For all three systems, the H_2_O_2_ concentration gradually approached a plateau after approximately 35 min of ultrasonication. Furthermore, when the dissolved O_2_ concentration was systematically adjusted by Ar/O_2_ bubbling (Figure S5), both CSPs and YSPs exhibited an approximately linear increase in H_2_O_2_ production with increasing dissolved O_2_ concentration (Figure 1d). Notably, the enhancement induced by increasing dissolved O_2_ was more pronounced for YSPs than for CSPs. At a dissolved O_2_ concentration of 12 mg L^−1^ and after 1 h of ultrasonication, the H_2_O_2_ yield of YSPs was approximately six times higher than that of CSPs (Figure 1d).

These results demonstrate the essential role of dissolved O_2_ in sonochemical H_2_O_2_ generation and highlight the superior performance of YSPs in facilitating H_2_O_2_ production. The enhanced activity of YSPs originates from their unique yolk–shell architecture, where the hydrophobic PTFE yolks remain accessible to the surrounding aqueous environment through the internal void space, while the hydrophilic PEM shells maintain particle dispersibility. In contrast, the PTFE surfaces in CSPs are partially shielded by the PEM coating and are accessible only through permeation pathways within the shell, limiting the effective PTFE–water interfacial area. The structural differences between YSPs and CSPs were further reflected during prolonged ultrasonication under oxygen‐rich conditions. YSPs gradually migrated to the water surface due to gas bubble formation and retention at the exposed hydrophobic PTFE interfaces (Figure 1d and Figure S6), whereas CSPs remained dispersed in water (Figure S7). Nevertheless, the hydrophilic PEM shells enabled the floated YSPs to be readily redispersed by gentle shaking, allowing continuous H_2_O_2_ generation with negligible loss of activity.

Owing to their excellent aqueous dispersibility, YSPs were further employed for in situ EPR characterization of reactive species generated during ultrasonication (Figure 1e). After 30 min of ultrasonication, distinct signals corresponding to HO^•^ and O_2_^•−^ were detected in O_2_‐saturated aqueous dispersions of YSPs. In contrast to deaerated conditions, where the O_2_^•−^ signal was negligible, only a weak HO^•^ signal was observed, and no measurable H_2_O_2_ accumulated; pronounced characteristic EPR signals for both HO^•^ and O_2_^•−^ rapidly emerged upon increasing the O_2_ concentration to 5 mg L^−1^ (further to 10 mg L^−1^), accompanied by significant H_2_O_2_ accumulation. These results indicate that ultrasound irradiation can generate HO^•^ through water activation even without dissolved O_2_, whereas HO^•^ recombination alone is insufficient for H_2_O_2_ accumulation. The emergence of O_2_^•−^ together with increased H_2_O_2_ production in the presence of dissolved O_2_ demonstrates that the oxygen‐dependent reduction pathway plays a dominant role in H_2_O_2_ generation.

**SCHEME 1:**
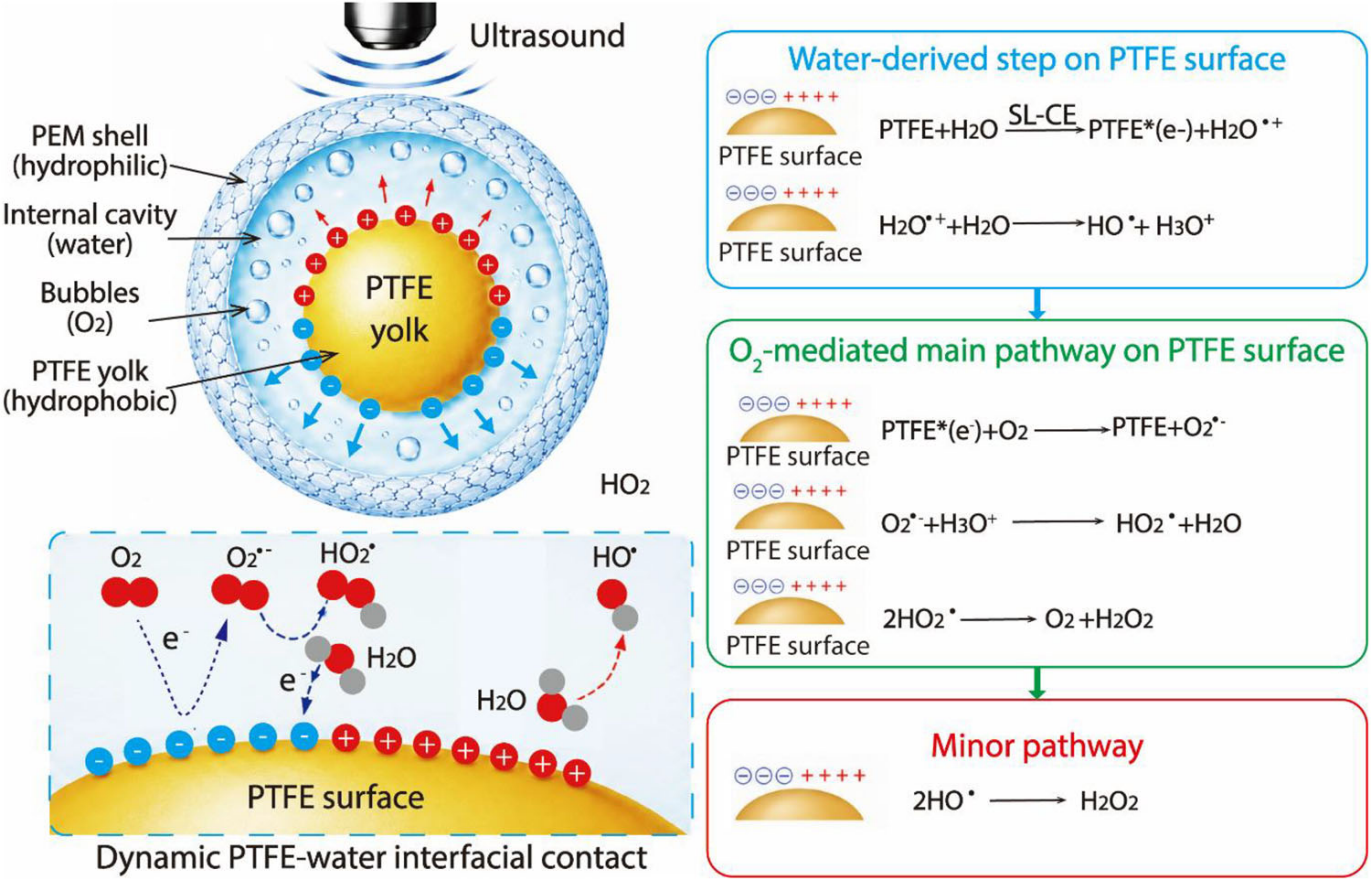
Mechanistic illustration of ultrasound‐driven interfacial H_2_O_2_ generation by PEM‐caged PTFE yolk–shell particles. The PEM shell provides aqueous dispersibility while maintaining accessibility of the PTFE surface. Ultrasonic‐induced PTFE–water contact electrification generates interfacial charges, enabling O_2_ reduction to O_2_^•−^/HO_2_^•^ intermediates and subsequent H_2_O_2_ formation.

On the basis of the EPR results and previously reported contact‐electrification mechanisms [22], an ultrasound‐driven interfacial reaction process is proposed (Scheme 1, see details in Supporting Information). Under acoustic cavitation, dynamic solid–liquid contact separation induces contact electrification at the PTFE–water interface, driving coupled oxygen reduction and water oxidation pathways.

1. Dominant Oxygen‐Reduction Pathway (O_2_‐Driven, Main Route): Dissolved O_2_ serves as the essential electron acceptor. Through solid–liquid contact electrification (SL‐CE), electrons accumulated on the perfluorinated PTFE surface, denoted as PTFE*(e^−^) reduce dissolved O_2_ to superoxide radical anions (O_2_^•−^, Equation S6), which rapidly protonate to hydroperoxyl radicals (HO_2_^•^, Equation S7). These oxygen‐derived intermediates subsequently convert to H_2_O_2_ via self‐dismutation or proton‐coupled reduction (Equation S8) [22].
2. Auxiliary Water‐Oxidation Pathway (Minor Contribution): Concurrently, water molecules donate electrons to PTFE to form water radical cations (H_2_O^•+^, Equation S4), which react with neighboring water molecules to yield hydroxyl radicals (HO^•^, Equation S5). However, the direct recombination of two HO^•^ radicals yield only a negligible amount of H_2_O_2_ (Equation S9) [23].

The indispensable role of dissolved O_2_ is directly evidenced by the complete absence of measurable H_2_O_2_ under degassed (low‐O_2_) conditions, providing that HO^•^ recombination alone is insufficient for effective H_2_O_2_ production. The yolk–shell architecture preserves high exposure of perfluorinated PTFE surfaces to both H_2_O and dissolved O_2_, thereby accelerating interfacial charge transfer and O_2_‐driven H_2_O_2_ synthesis relative to CSPs.

The substantially stronger EPR signals observed under O_2_‐saturated conditions are consistent with this proposed mechanism and further support the structural advantage of the YSPs. Their yolk–shell architecture preserves extensive accessibility of the perfluorinated PTFE surfaces to both H_2_O and dissolved O_2_, thereby promoting dynamic solid–liquid contact, interfacial charge transfer, and the generation of reactive oxygen intermediates under ultrasonication. These interfacial processes collectively account for the enhanced ultrasound‐driven H_2_O_2_ production of YSPs relative to CSPs. To obtain deeper quantitative insight into the structural factors governing this enhancement, we subsequently varied the size and surface chemistry of the PTFE yolks.

### 2.3 Quantitative Assessment of Size and Surface Nature Effects on YSP‐facilitated Sonochemical H_2_O_2_ Generation

**FIGURE 2:**
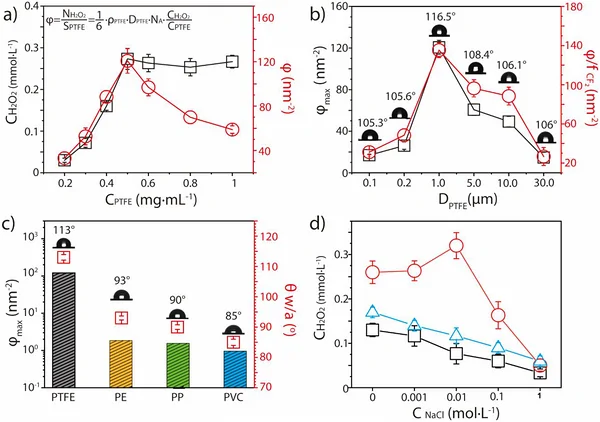
Quantitative assessment of YSP‐facilitated sonochemical H_2_O_2_ generation. (a) Plots of $C_{H_{2}O_{2}}$ generated in aqueous dispersion of YSPs bearing 1 µm PTFE yolks after 1 h ultrasonication (squares) and the corresponding φ (circles) versus the mass concentration of PTFE yolks (*C_PTFE_*) of the YSPs. (b) Plot of the φ_*max*_ (squares) and $f_{CF_{2}}$ (circles) of as‐prepared YSPs versus the *D_PTFE_* of their PTFE yolks. The photos of water droplets (2 µL) on the plates of densely compressed YSPs in air are inset with corresponding water contact angles marked. The φ_*max*_ values are obtained from the data shown in Figure S9. (c) Summary of plot of the φ_*max*_ values of YSPs bearing PTFE, PE, PP, and PVC yolks with an average size of 1 µm and the water‐in‐air contact angle (θ_*w*/*a*_) of the corresponding densely compressed YSP plates. The photos of water droplets (2 µL) on the YSP plates in air are inset. The φ_*max*_ values are obtained from the data shown in Figure S13. In Figure 2a–c, sonochemical H_2_O_2_ generation is implemented by 1 h ultrasonication of 10 mL of aqueous dispersion of YSPs at pH 6, in which the *C_PTFE_* values of YSPs can be found in Figure 2a and Figure S8. (d) Plots of $C_{H_{2}O_{2}}$ in saline dispersion of YSPs at pH of 3 (squares), 6 (circles), and 9 (triangles) obtained after 1 h of ultrasonication versus NaCl concentration (*C_NaCl_*).

The power of YSPs to substantially promote sonochemical H_2_O_2_ generation stems from their peculiar yolk–shell structures, in which liberated PTFE particles are caged in PEM capsules, thereby ensuring flexible dispersion of PTFE yolk in aqueous media at designated tunable concentrations and, at the same time, all CF_2_ moieties on their surfaces are in direct contact with aqueous surroundings. Distinct from pristine PTFE particles as well as other hydrophilic particles with negligible dispersibility in water, these YSPs enable us to systematically and quantitatively interrogate the effects of their structural and chemical features on sonochemical H_2_O_2_ generation (Figure S8). As shown in Figure 2a and Figure S9, the concentration of H_2_O_2_ ($C_{H_{2}O_{2}}$) generated in aqueous dispersions of YSPs during ultrasonication increases with PTFE yolk concentration (*C_PTFE_*) and reaches a plateau $C_{H_{2}O_{2}}$ of 0.27 mmol·L^−1^ at *C_PTFE_* of 0.5 mg·mL^−1^. To gain in‐depth insight to the revealed $C_{H_{2}O_{2}}$ – *C_PTFE_* correlation, here the efficiency of YSPs to facilitate H_2_O_2_ production is defined as the number of H_2_O_2_ generated on individual YSPs ($N_{H_{2}O_{2}}$) per PTFE yolk surface area (*S_PTFE_*) – φ (nm^−2^) – according to Equation (1):

(1) $$\varphi =\frac{N_{H_{2}O_{2}}}{S_{PTFE}}=\frac{1}{6}\cdot \rho_{PTFE}\cdot D_{PTFE}\cdot N_{A}\cdot \frac{C_{H2O2}}{C_{PTFE}}$$

where ρ_*PTFE*_ is the PTFE density (2.2 g·cm^−3^), *D_PTFE_* is the diameter of the PTFE yolk and *N_A_* is Avogadro's number. For a given *D_PTFE_*, YSPs show a volcano‐shaped dependence of *φ* on *C_PTFE_* (Figure 2a and Figure S9). The presence of optimal *C_PTFE_* to achieve φ maximum (φ_*max*_) reflects an delicate balance between O_2_ consumption and dissolution in water during YSP‐facilitated sonochemical H_2_O_2_ generation, since the limited solubility of O_2_ in water (Figure S5) significantly constrains the utilization of YSPs – precisely speaking, the hydrophobic surface area of their PTFE yolks – particularly at high *C_PTFE_*. Figure 2b reveals a volcano‐shaped dependence of the φ_*max*_ of YSPs on *D_PTFE_* – 1 µm PTFE yolks exhibit the highest φ_*max*_ (∼120 nm^−2^), 6 times as large as those of 0.1 and 30 µm counterparts – and it is fairly consistent with the surface hydrophobicity, measured as water contact angle in air (Figure S10), and in turn the surface coverage of CF_2_ groups ($f_{CF_{2}}$) on PTFE yolks (Figure S11).^1^ This establishes an unambiguous correlation between the ability of YSPs, namely, PTFE yolks, to facilitate sonochemical H_2_O_2_ generation and the perfluorinated surface chemistry.

In order to gain a better understanding of the role of hydrophobic surfaces in facilitating sonochemical H_2_O_2_ generation, here we applied PAH/PSS PEM coatings on other hydrophobic particles such as polyethylene (PE), polypropylene (PP), and poly(vinyl chloride) (PVC) instead of PTFE particles to construct YSPs. While all hydrophobic yolks enable sonochemical H_2_O_2_ generation, as shown in Figure 2c and Figures S12 and S13, fluoro‐free yolks lead to substantially lower production yield with maximal $C_{H_{2}O_{2}}$ of 0.054 mmol·L^−1^, approximately 5 times lower than that of PTFE yolks (0.27 mmol·L^−^^[^ ^1^ ^]^) and φ_*max*_ in range of 1–2 nm^−2^, approximately 100 times lower than that of PTFE yolks (∼120 nm^−2^). It is worth noting that the φ_*max*_ of PE, PP, and PVC yolks notably decreases with their water contact angle (θ_*w*/*a*_) decreasing and in turn surface hydrophobicity decreasing.

### 2.4 Effects of pH and Salt Concentration on YSP‐Facilitated Sonochemical H_2_O_2_ Generation

Here we also explored the effects of pH and salt concentration on sonochemical H_2_O_2_ generation in aqueous dispersion of YSPs, since ions, especially OH^−^ and Cl^−^ anions, are known for their propensity to adsorb onto hydrophobic surfaces in water and in turn reduce the surface hydrophobicity [24]. Figure 2d shows that YSP‐facilitated sonochemical H_2_O_2_ generation is noticeably less effective in acidic or basic water than in neutral water, and its production yield decreases with NaCl concentration, confirming the negative impact of ion adsorption on the surface hydrophobicity of PTFE yolks and thus on their ability to facilitate cavitation in water under ultrasonication. These results offer direct experimental testimony not only to an essential role of hydrophobic yolks in YSP‐facilitated sonochemical H_2_O_2_ generation but also to the high efficiency of fluorinated surfaces as low‐surface‐energy platforms to facilitate cavitation in water under ultrasonication and, in turn, decomposition of H_2_O and O_2_ into highly reactive HO^•^ and O_2_^•−^ radicals. Furthermore, YSPs achieve the highest sonochemical H_2_O_2_ production yields in neutral water at a NaCl concentration of 10 mM (Figure 2d), reflecting that water dissociation can be effectively enhanced in the presence of NaCl at fairly low concentrations (on the order of mM), thus facilitating water decomposition under ultrasonication.

### 2.5 Stability and Reaction Kinetics of YSPs for Sonochemical H_2_O_2_ Generation

**FIGURE 3:**
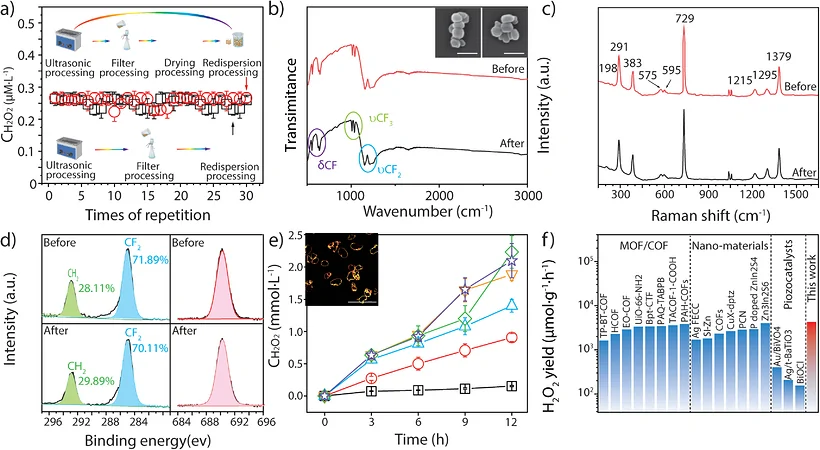
Stability and reusability of YSPs for sonochemical H_2_O_2_ generation. (a) Plots of $C_{H_{2}O_{2}}$ in aqueous dispersions of YSPs (0.5 mg·mL^−1^) under ultrasonication versus the times of reuse cycles. The cycle of YSP reuse is implemented by either straightforwardly redispersing the YSPs, collected after 1 h of ultrasonication, into water (red circles) or drying the collected YSPs before redispersion (black squares), schematically illustrated in the insets. (b, c) Fourier transform infrared spectra (b) and Raman spectra (c) of YSPs before and after 12 h of ultrasonication in water, where characteristic vibrational bands assigned to CF_2_ and CH_2_ are marked. Scanning electron micrographs of YSPs obtained before and after 12 h of ultrasonication are inset in Figure b. (d) The high‐resolution XPS spectra of carbon (left) and fluorine (right) of YSPs obtained before and after 12 h of ultrasonication in water. The corresponding full XPS spectra are shown in Figure S15. (e) Plots of $C_{H_{2}O_{2}}$ versus ultrasonication time during sonochemical H_2_O_2_ generation in water bearing YSPs at concentrations of 0.02 (squares), 0.04 (circles), 0.06 (upward triangles), 0.08 (downward triangles), 0.1 (diamonds), and 0.12 mg·mL^−1^ (stars). The YSPs used in all Figures bear 1 µm PTFE yolks. (f) Comparison of H_2_O_2_ generation with photocatalysis and piezocatalysis [17, 18, 19, 25, 26, 27, 28, 29, 30, 31, 32, 33, 34, 35, 36, 37, 38, 39] (Table S1).

It is worth noting that all intermediate species such as HO^•^ and O_2_^•−^ radicals and the end product – H_2_O_2_ –formed in water under ultrasonic irradiation are rather reactive and oxidative. The present design of YSPs composed of PTFE yolks caged in PAH/PSS PEM shells may benefit from the fact that the potential risk of oxidation of the hydrocarbon backbones of PAH and PSS, as well as oxidation of PAH's amine groups into nitrate groups, may de facto increase the shell hydrophilicity, while the prominent chemical inertness of PTFE yolks ensure their surface hydrophobicity stable during sonochemcial H_2_O_2_ generation. After 12 h of more intense ultrasonication in water, YSPs exhibit virtually no change in surface morphology and chemistry; negligible loss in the surface coverage of CF_2_ (for PTFE yolks) and CH_2_ (for PAH/PSS PEM shells) is observed (Figures 3b–d, S14, and S15). Since YSPs can be readily filtered out of aqueous media, furthermore, their reusability was also tested against repeated use for sonochemical H_2_O_2_ generation. Note that the collected YSPs were either directly redispersed in fresh water or submitted to thorough drying to intentionally induce particle aggregation before redispersion. Figure 3a unveils negligible change in H_2_O_2_ production yield after YSPs are repeatedly used over 30 cycles; 1 h of ultrasonication per cycle. This demonstrates not only the chemical stability of YSPs against repetitive use for sonochemical H_2_O_2_ generation thanks to the inertness of their PTFE yolks but also their colloidal stability against drying‐induced aggregation thanks to the PEM shells.

Taking advantage of their excellent dispersibility in water and chemical stability against ultrasonication treatment, the present YSPs enabled quantitative assessment of the reaction kinetics of sonochemical H_2_O_2_ generation in water, which is rarely touched on in the literature due to the negligible dispersibility of hydrophobic particles in water. Figure 3e shows that $C_{H_{2}O_{2}}$ increases progressively with ultrasonication time and reaches the plateau values acfter 12 h of ultrasonication, and the plateau $C_{H_{2}O_{2}}$ values initially increase with *C_PTFE_* and become hardly changed after *C_PTFE_* is set above 0.08 mg·mL^−1^; the maximal $C_{H_{2}O_{2}}$ values is 2.5 mM after 12 h of ultrasonication (1000 W). As such, the maximal production rate of YSP‐facilitated sonochemical H_2_O_2_ generation is achieved at a YSP concentration of 0.06 mg·mL^−1^, and it is as high as 3.34 mmol∙g_cat_^−1^∙h^−1^ (3340 µmmol·g_cat_^−1^·h^−1^), significantly surpassing the performance of hydrophobic particle‐assisted sonochemical H_2_O_2_ generation and well matching to the production rates of photocatalytic H_2_O_2_ generation methods in the absence of sacrificial agents reported in the literature. (Figure 3f and Table S1) The remarkable performance of YSPs prompted us to explore their practical utility for degrading organic pollutants in water.

### 2.6 The Practical Applications of YSPs‐Facilitated Sonochemical H_2_O_2_ Generation

**FIGURE 4:**
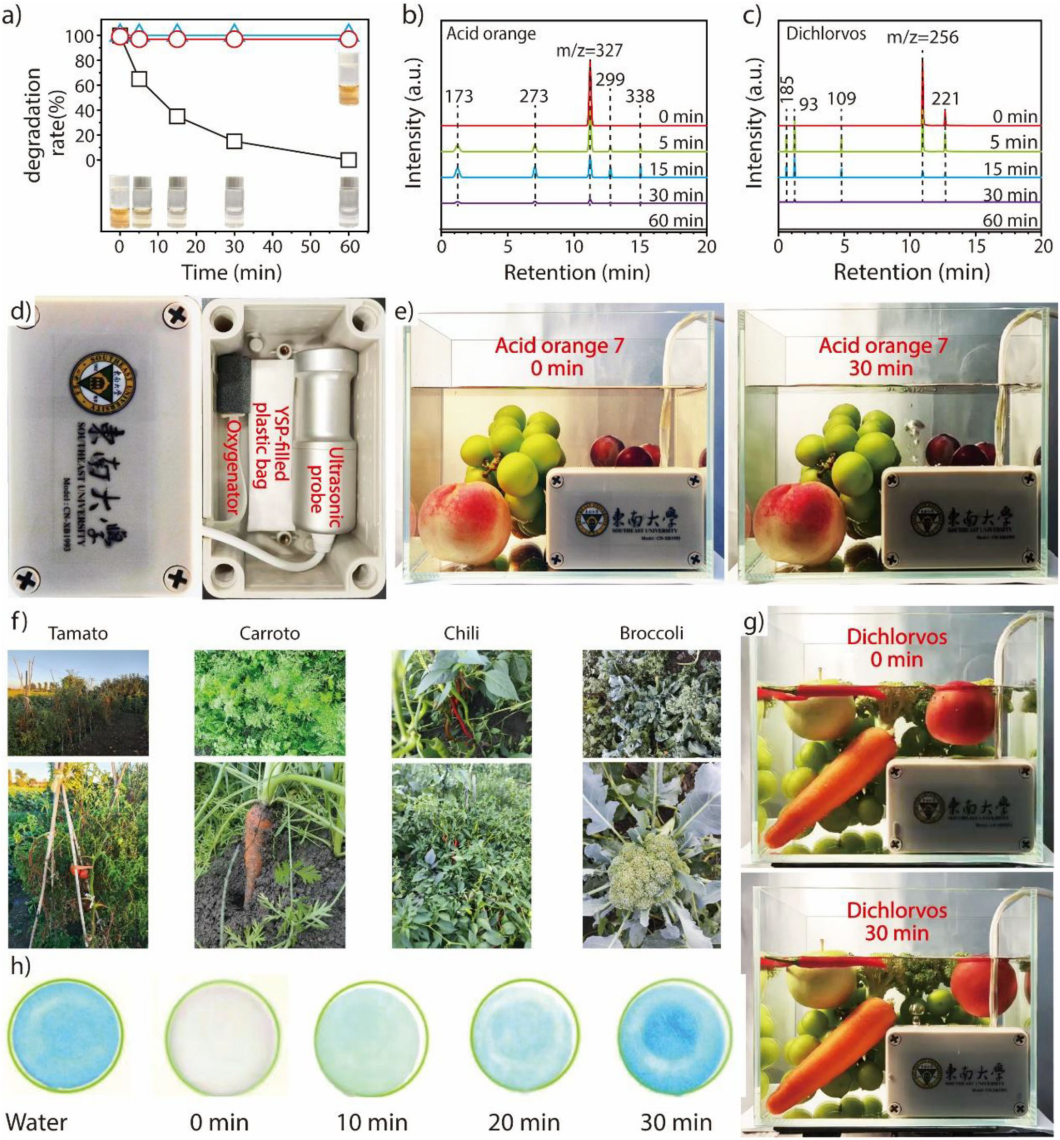
YSP‐facilitated sonochemical degradation of organic pollutants in water. (a) Plots of the degradation degree of AO7 in water in the presence of YSPs (1 mg mL^−1^) under ultrasonic irradiation (squares) versus the treatment time. For comparison, the results obtained without ultrasonication treatment (circles) and without the use of YSPs under ultrasonic irradiation (triangles) are shown. (b, c) LC‐MS data of aqueous solutions of AO7 (b) and dichlorvos (c) in the presence of YSPs (1 mg mL^−1)^ under ultrasonic irradiation for 0, 5, 15, 30, and 60 min. (d) Photos of a home‐built YSP‐facilitated H_2_O_2_ generator, with dimensions of 80 × 130 × 70 mm. Key components, including a custom‐configured ultrasonic probe (60 kHz, 100 W), a YSP‐filled plastic bag, and an oxygenator, are marked. (e) Photos of a water tank with a home‐built YSP‐facilitated H_2_O_2_ generator placed inside, in which peach, grapes, and cherries with AO7 molecules deliberately adsorbed on their surfaces are readily cleaned up after the H_2_O_2_ generator is switched on for 30 min, evidenced by the disappearance of orange color in water due to the release of adsorbed dyes from the fruit surfaces. (f, g) Photos of freshly collected tomato, carrot, chilli and broccoli with surfaces intentionally sprayed with dichlorvos (f), which are cleansed by a home‐built YSP‐facilitated H_2_O_2_ generator placed in a water tank (g). (h) Optical images of commercial organophosphorus pesticide test kits using water samples collected from vegetables treated with the homemade H_2_O_2_ generator for 0–30 min. After the H_2_O_2_ generator is switched on for 0, 10, 20, and 30 min, aliquots of water is taken out from the tank, and the white core of the test paper is a sign of the presence of pesticides in water, which is readily turned to greenish and eventually to blue after 30 min ultrasonication, comparable to that observed using clean water, indicating the complete degradation of pesticides.

H_2_O_2_ is widely deployed for oxidative degradation of organic pollutants in aqueous environments due to its high efficiency and environmentally benign byproducts – O_2_ and H_2_O [40, 41]. In this context, here we assessed the applicability of YSPs‐facilitated sonochemical H_2_O_2_ generation to break down organic pollutants in aqueous media. After YSPs are introduced in aqueous solution of model organic pollutants, acid orange 7 (AO7), as shown in Figure 4a, the dye concentration in water is rapidly reduced from 5 mg·L^−1^ to < 50 µg·L^−1^ (50 ppb) after 60 min of ultrasonication, meeting WHO safety standards for drinking water [42]; no dye degradation is accomplished without YSPs introduced in aqueous dye solutions or without ultrasonic irradiation (Figure S16). Note that ultrasonication of the ethanol solution of AO7 in the presence of YSPs leads to a non‐negligible change in dye concentration, underlining that the dyes are decomposed by H_2_O_2_ in situ formed in water under ultrasonic radiation in the presence of YSPs (Figure S17). Liquid chromatography – mass spectroscopy (LC‐MS) analysis confirms complete degradation of AO7 molecules with negligible organic residues in water after 60 min of ultrasonication. (Figure 4b and Table S2) Furthermore, the present YSP‐facilitated sonochemical treatment enables effective decomposition of dichlorvos – widely used, commercial organophosphorus pesticide – in water; 60 min of ultrasonication in the presence of 1 mg·mL^−1^ YSPs is sufficient to completely clean up water from 1 ppm dichlorvos (1 mg·L^−1^) with negligible organic residuals, as evidenced by LC‐MS analysis (Figure 4c and Table S3).

Encouraged by these results, a prototype YSP‐facilitated H_2_O_2_ generator was devised for household applications (Figure 4d). This home‐built apparatus (80 × 130 × 70 mm) integrates a custom‐configured ultrasonic probe operating at 60 kHz with a power output of 100 W, where a plastic bag filled with YSPs is juxtaposed with an oxygenator to ensure a continuous supply of O_2_ during sonochemical H_2_O_2_ generation in water. (see Figure S18 for details). As shown in Figure 4e, this device can readily clean up fruits of AO7 molecules adsorbed on their surfaces after it is switched on for 30 min; the dye molecules are completely decomposed, with no residual dye molecules detected in the water phase. To test its practical utility, as shown in Figure 4f–h, freshly collected vegetables are intentionally sprayed with an aqueous solution of dichlorvos (Figure 4f), which leaves behind a substantial amount of pesticide residuals on the vegetable surfaces, especially the complex flowerhead geometry of broccoli, which comprises tiny buds and branching structures, after being simply rinsed with fresh tap water. When the pesticide‐contaminated vegetables are immersed in a water tank in the presence of a YSP‐facilitated H_2_O_2_ generator, all pesticide remaining is completely degraded with no organic residues detected after the generator is switched on for 30 min (Figure 4g,h), thus highlighting the high cleaning efficiency of the present H_2_O_2_ generator and its operational convenience for decentralized, small‐scale H_2_O_2_ application in household settings.

## 3 Conclusions

In summary, this work reports an innovative yolk–shell structure composed of hydrophobic PTFE particles fully caged within hydrophilic PEM shells, which enables direct dispersion of the PTFE particles in water without alteration of their perfluorinated surface chemistry. Thanks to their direct contact with aqueous media, the PTFE yolks can significantly facilitate interfacial cavitation on their hydrophobic surfaces, thereby driving the effective conversion of H_2_O and O_2_ into highly reactive HO^•^ and O_2_^•−^ radicals. As such, this yolk–shell structure allows us not only to harness water‐immiscible PTFE particles (yolks) for catalyst‐free, sonochemical H_2_O_2_ generation with a yield of 2.5 mM over 12 h – corresponding to a production rate as high as 3.34 mmol∙g_cat_^−1^∙h^−1^, comparable to that of state‐of‐the‐art organic photocatalytic systems in the absence of sacrificial agents – but also quantitatively elucidate and design the promoting performance of PTFE particles in sonochemical H_2_O_2_ generation according to their structural and surface chemical features. All these technical merits have not been achieved in previous studies of deploying PTFE particles for sonochemical H_2_O_2_ generation.

Since PEM shells grant PTFE yolks an excellent adaptability in aqueous media and a full, direct exposure to water, the present YSPs were utilized to convert commercial, portable ultrasonicators to user‐friendly H_2_O_2_ generators, which allow us to readily and rapidly degrade over 99.9% of organic pollutants such as dyes and pesticides left over on fruits or vegetables, thus paving innovative ways for small‐scale, on‐demand H_2_O_2_ applications particularly in household and clinical settings. It is important to stress that hydrophobic polymer fine particles, including PTFE, PE, PP, and PVC used in the present YSP system, are the processed products of polymer wastes and widely available in the market, and they can be easily recycled and reused. Bearing that in mind, the present YSP design for sonochemical generation of H_2_O_2_ – a versatile, eco‐friendly oxidant – promises an innovative transformation of adverse polymer wastes into advanced treatment tools for organic or biological contaminants, in particular for personal hygiene and public health, thus offering a significant contribution to the sustainability of our society development.

## 4 Methods

### 4.1 Constructing Hydrophilic Polyelectrolyte Multilayer (PEM) Coatings on Hydrophobic Polymer Particles to Obtain Hydrophobic Polymer/PEM Core–Shell Particles (CSPs)

Taking advantage of those hydrophobic particles' reasonable dispersibility and colloidal stability in degassed saline water, they were subjected to layer‐by‐layer (LbL) assembly of PAH and PSS according to our previous work [20].

Firstly, hydrophobic particles of polyethylene (PE), polypropylene (PP), polyvinyl chloride (PVC), and polytetrafluoroethylene (PTFE) were thoroughly cleaned with ethanol and water to remove any impurities from their surfaces. After air‐drying, a specific amount of particle powder was dispersed in a degassed aqueous solution of PAH (1.0 mg/mL) containing 1 M NaCl. The mixture was vigorously homogenized for 20 min. Subsequently, the hydrophobic particles were filtered and washed thoroughly with water to remove excess PAH and NaCl, respectively.

Next, the PAH‐coated particles were immersed in a degassed aqueous solution of PSS (1.0 mg/mL) containing 1 M NaCl and incubated for 20 min. After washing with water to remove excess PSS and NaCl, the particles were again immersed in a degassed aqueous solution of PAH (1.0 mg/mL) containing 1 M NaCl and incubated for 20 min.

This cycle of alternating PAH and PSS deposition was repeated m times to construct (PAH/PSS)_m_ shells on the target hydrophobic particles, resulting in hydrophobic core/hydrophilic shell particles.

### 4.2 Preparation of Hydrophobic Polymer/PEM Yolk–Shell Particles (YSPs)

After forming (PAH/PSS)_m_ polyelectrolyte multilayers (PEM) shells with a specific bilayer number (m), the obtained hydrophobic polymer/hydrophilic PEM core–shell particles (CSPs) were incubated in aqueous solution of 0.1 M hydrochloric acid solution for 6 h. Subsequently, the particles were neutralized with a 0.01 M NaOH solution to remove any residual HCl. And then, they were washed repeatedly with deionized water until the conductivity of the filtrate matched that of pure water. Finally, the particles were redispersed in an aqueous medium.

This cycle, involving incubation in 0.1 M HCl aqueous solution, neutralization with 0.01 M NaOH aqueous solution, filtration, and redispersion in deionized water, was repeated five times to make sure that the hydrophobic interactions between the PEM shells and the hydrophobic cores were totally cleaved, liberating the cores and yielding hydrophobic polymer/PEM yolk–shell particles (YSPs).

## Author Contributions

**Chongling Cheng**: conceptualization, funding acquisition, writing – original draft, writing – review and editing, visualization, supervision, project administration. **Dayang Wang**: writing – review and editing, funding acquisition, supervision, conceptualization, methodology, project administration, resources. **Rong Wang**: investigation, validation, software, formal analysis, writing – original draft, data curation. **Yue Yang**: methodology, data curation. **Bo Wang**: methodology, software, data curation, formal analysis, validation, investigation. **Zhenhui Qi**: funding acquisition, writing – review and editing, visualization. **Tengyue Fu**: methodology, data curation.

## Conflicts of Interest

The authors declare no conflicts of interest.

## Supporting information




**Supporting File**: advs77281‐0001‐SuppMat.pdf.

## Data Availability

The data that supports the findings of this study are available in the supplementary material of this article.

## Endnote

^1^This Notable Variation in f_CF2_ is Assigned to Different Processing Methods Used to Produce Commercial PTFE Particles.

## References

[1] C. W. Jones , Application of Hydrogen Peroxide and Derivatives (Royal Society of Chemistry, 1999), 10.1039/9781847550132.

[2] G. Strukul , ed., Catalytic Oxidations With Hydrogen Peroxide as Oxidant (Springer, 1992), 10.1007/978-94-017-0984-2.

[3] X. Zhang , X. Yang , B. Su , et al., “Membrane Electrode Assembly for Hydrogen Peroxide Electrosynthesis,” Nature Reviews Clean Technology 1, no. 6 (2025): 413–431, 10.1038/s44359-025-00069-7.

[4] X. Zou , Q. Shi , M. Cheng , et al., “Metal–Organic Framework‐Based Materials for Photocatalytic Hydrogen Peroxide Production: Insights Into Mechanism, Modification Strategies, and Environmental Applications,” Advanced Energy Materials 15, no. 30 (2025): 2501424, 10.1002/aenm.202501424.

[5] S. C. Perry , D. Pangotra , L. Vieira , et al., “Electrochemical Synthesis of Hydrogen Peroxide From Water and Oxygen,” Nature Reviews Chemistry 3, no. 7 (2019): 442–458, 10.1038/s41570-019-0110-6.

[6] G. Yu , et al., “Electrochemical Synthesis of Hydrogen Peroxide Enabled by Tri‐Coordinated Cobalt Sites in Silicate‐1 Zeolite,” Angewandte Chemie International Edition 64 (2025): 202506390. 10.1002/anie.202506390 40219623

[7] S. Lin , J. Wang , J. Chen , et al., “Electrochemical Pilot H_2_O_2_ Production by Solid‐State Electrolyte Reactor: Insights From a Hybrid Catalyst for 2‐Electron Oxygen Reduction Reaction,” Angewandte Chemie International Edition 64, no. 19 (2025): 202502144, 10.1002/anie.202502144. 40033944

[8] H. Hou , X. Zeng , and X. Zhang , “Production of Hydrogen Peroxide by Photocatalytic Processes,” Angewandte Chemie International Edition 59, no. 40 (2020): 17356–17376, 10.1002/anie.201911609. 31571331

[9] L. Li , Q. Bu , T. Lang , et al., “Leveraging Carbon Quantum Dot Spacers as Dual Proton/Electron Boosters to Upgrade TiO_2_@Polydopamine Photocatalysts for pH‐Resilient Conversion of O_2_ into H_2_O_2_ ,” Angewandte Chemie International Edition 64, no. 16 (2025): 202501357, 10.1002/anie.202501357. 39915254

[10] J. K. Lee , K. L. Walker , H. S. Han , et al., “Spontaneous Generation of Hydrogen Peroxide From Aqueous Microdroplets,” Proceedings of the National Academy of Sciences 116, no. 39 (2019): 19294–19298, 10.1073/pnas.1911883116. PMC6765303 31451646

[11] K. Brudzynski , “A Current Perspective on Hydrogen Peroxide Production in Honey. A Review,” Food Chemistry 332 (2020): 127229, 10.1016/j.foodchem.2020.127229. 32688187

[12] Z. Wang , A. Berbille , Y. Feng , et al., “Contact‐Electro‐Catalysis for the Degradation of Organic Pollutants Using Pristine Dielectric Powders,” Nature Communications 13, no. 1 (2022): 130, 10.1038/s41467-021-27789-1. PMC8748705 35013271

[13] L. E. Krushinski and J. E. Dick , “Direct Electrochemical Evidence Suggests That Aqueous Microdroplets Spontaneously Produce Hydrogen Peroxide,” Proceedings of the National Academy of Sciences 121, no. 12 (2024): 2321064121, 10.1073/pnas.2321064121. PMC10962973 38466847

[14] Y. Wang , Y. Xu , S. Dong , et al., “Ultrasonic Activation of Inert Poly(tetrafluoroethylene) Enables Piezocatalytic Generation of Reactive Oxygen Species,” Nature Communications 12, no. 1 (2021): 3508, 10.1038/s41467-021-23921-3. PMC8190189 34108484

[15] J. Zhao , X. Zhang , J. Xu , W. Tang , Z. Lin Wang , and F. Ru Fan , “Contact‐electro‐catalysis for Direct Synthesis of H_2_O_2_ Under Ambient Conditions,” Angewandte Chemie International Edition 62, no. 21 (2023): 202300604, 10.1002/anie.202300604. 36949023

[16] G. J. Puts , P. Crouse , and B. M. Ameduri , “Polytetrafluoroethylene: Synthesis and Characterization of the Original Extreme Polymer,” Chemical Reviews 119, no. 3 (2019): 1763–1805, 10.1021/acs.chemrev.8b00458. 30689365

[17] Z. Gao , F. Liu , Z. Chen , et al., “Defect‐Modulated Oxygen Adsorption and Z‐Scheme Charge Transfer for Highly Selective H_2_O_2_ Photosynthesis in Pure Water,” Nature Communications 16, no. 1 (2025): 8889, 10.1038/s41467-025-64166-8. PMC12504443 41057375

[18] W. Chi , J. Wu , Y. Dong , J. Wu , and Y. Zhu , “Accelerated Exciton Dissociation and Charge Transfer via Third‐Motif Engineered Conjugated Polymers for Photocatalytic Circulation‐Flow Synthesis of H_2_O_2_ ,” Angewandte Chemie International Edition 64 (2025): 202508690. 10.1002/anie.202508690 40465429

[19] X. Chi , Z. Zhang , M. Li , et al., “Vinylene‐Linking of Polycyclic Aromatic Hydrocarbons to π‐Extended Two‐Dimensional Covalent Organic Framework Photocatalyst for H_2_O_2_ Synthesis,” Angewandte Chemie International Edition 64, no. 7 (2024): 202418895, 10.1002/anie.202418895. 39406685

[20] B. Wang , T. Fu , C. Cheng , and D. Wang , “Layer‐by‐Layer Assembly of Polyelectrolytes on Hydrophobic Particles in Aqueous Milieu for Efficient Dye Removal,” Chemical Communications 59, no. 83 (2023): 12479–12482, 10.1039/D3CC04149B. 37782535

[21] A. P. R. Johnston , C. Cortez , A. S. Angelatos , and F. Caruso , “Layer‐by‐Layer Engineered Capsules and Their Applications,” Current Opinion in Colloid & Interface Science 11, no. 4 (2006): 203–209, 10.1016/j.cocis.2006.05.001.

[22] A. Berbille , X.‐F. Li , Y. Su , et al., “Mechanism for Generating H_2_O_2_ at Water‐Solid Interface by Contact‐Electrification,” Advanced Materials 35, no. 46 (2023): 2304387, 10.1002/adma.202304387. 37487242

[23] B. Chen , Y. Xia , R. He , et al., “Water–Solid Contact Electrification Causes Hydrogen Peroxide Production From Hydroxyl Radical Recombination in Sprayed Microdroplets,” Proceedings of the National Academy of Sciences 119, no. 32 (2022): 2209056119, 10.1073/pnas.2209056119. PMC9371641 35914139

[24] D. Horinek and R. R. Netz , “Specific Ion Adsorption at Hydrophobic Solid Surfaces,” Physical Review Letters 99, no. 22 (2007): 226104, 10.1103/PhysRevLett.99.226104. 18233302

[25] Z.‐Z. Liang , Y. Wang , X.‐A. Li , et al., “Enhanced Piezo‐Photocatalytic Water Splitting Activity via Engineering Robust Dipole Moments in Covalent Organic Frameworks,” Nature Communications 17, no. 1 (2026): 729, 10.1038/s41467-025-67467-0. PMC12819517 41387708

[26] Q. Zhu , L. Shi , Z. Li , G. Li , and X. Xu , “Protonation of an Imine‐linked Covalent Organic Framework for Efficient H_2_O_2_ Photosynthesis Under Visible Light up to 700 nm,” Angewandte Chemie International Edition 63, no. 32 (2024): 202408041, 10.1002/anie.202408041. 38738797

[27] P. Li , F. Ge , Y. Yang , et al., “1D Covalent Organic Frameworks Triggering Highly Efficient Photosynthesis of H_2_O_2_ via Controllable Modular Design,” Angewandte Chemie International Edition 63, no. 12 (2024): 202319885, 10.1002/anie.202319885. 38298054

[28] C. Wu , Z. Teng , C. Yang , et al., “Polarization Engineering of Covalent Triazine Frameworks for Highly Efficient Photosynthesis of Hydrogen Peroxide From Molecular Oxygen and Water,” Advanced Materials 34, no. 28 (2022): 2110266, 10.1002/adma.202110266. 35524761

[29] H. Xu , Y. Wang , Y. Xu , et al., “Integrating Multipolar Structures and Carboxyl Groups in sp^2^ ‐Carbon Conjugated Covalent Organic Frameworks for Overall Photocatalytic Hydrogen Peroxide Production,” Angewandte Chemie International Edition 63, no. 41 (2024): 202408802, 10.1002/anie.202408802. 39039037

[30] Q. Liu , H. Bi , R. Zhao , X. Yang , F. Chen , and Z. Shen , “Fully Exposed Silver Clusters Enabling Highly Efficient Photocatalytic H_2_O_2_ Production in Pure Water,” Angewandte Chemie International Edition 64, no. 37 (2025): 202511687, 10.1002/anie.202511687. 40685675

[31] Y.‐X. Ye , J. Pan , F. Xie , et al., “Highly Efficient Photosynthesis of Hydrogen Peroxide in Ambient Conditions,” Proceedings of the National Academy of Sciences 118, no. 16 (2021): 2103964118, 10.1073/pnas.2103964118. PMC8072241 33853952

[32] C. Wang , S. Tu , F. Chen , T. Ma , and H. Huang , “Asymmetric Single‐Unit‐Cell Layer Enriching Polar Inherent Hydroxyls Eliminates Interlayer Electric Field Shielding Effect and In Situ Self‐Polarize for Piezocatalytic Water Splitting,” Advanced Materials 37, no. 33 (2025): 2505592, 10.1002/adma.202505592. 40420679

[33] J. Zhang , H. Lei , Z. Li , F. Jiang , L. Chen , and M. Hong , “Halogen‐Modulated 2D Coordination Polymers for Efficient Hydrogen Peroxide Photosynthesis Under Air and Pure Water Conditions,” Angewandte Chemie International Edition 63, no. 3 (2024): 202316998, 10.1002/anie.202316998. 38017354

[34] S. Yan , Y. Li , X. Yang , X. Jia , J. Xu , and H. Song , “Photocatalytic H_2_O_2_ Generation Reaction With a Benchmark Rate at Air‐Liquid‐Solid Joint Interfaces,” Advanced Materials 36, no. 9 (2024): 2307967, 10.1002/adma.202307967. 37910074

[35] K. Zhang , L. Tian , J. Yang , et al., “Pauling‐Type Adsorption of O_2_ Induced by Heteroatom Doped ZnIn_2_S_4_ for Boosted Solar‐Driven H_2_O_2_ Production,” Angewandte Chemie International Edition 63, no. 5 (2024): 202317816, 10.1002/anie.202317816. 38082536

[36] J.‐L. Zhou , Y.‐F. Mu , M. Qiao , et al., “Unlocking One‐Step Two‐Electron Oxygen Reduction via Metalloid Boron‐Modified Zn_3_In_2_S_6_ for Efficient H_2_O_2_ Photosynthesis,” Angewandte Chemie International Edition 64, no. 28 (2025): 202506963, 10.1002/anie.202506963. 40317879

[37] Y. Wei , Y. Zhang , W. Geng , H. Su , and M. Long , “Efficient Bifunctional Piezocatalysis of Au/BiVO_4_ for Simultaneous Removal of 4‐Chlorophenol and Cr(VI) in Water,” Applied Catalysis B: Environmental 259 (2019): 118084, 10.1016/j.apcatb.2019.118084.

[38] J. Feng , Y. Fu , X. Liu , S. Tian , S. Lan , and Y. Xiong , “Significant Improvement and Mechanism of Ultrasonic Inactivation to Escherichia coli with Piezoelectric Effect of Hydrothermally Synthesized t‐BaTiO_3_ ,” ACS Sustainable Chemistry & Engineering 6, no. 5 (2018): 6032–6041, 10.1021/acssuschemeng.7b04666.

[39] D. Shao , L. Zhang , S. Sun , and W. Wang , “Oxygen Reduction Reaction for Generating H_2_O_2_ Through a Piezo‐Catalytic Process Over Bismuth Oxychloride,” Chemsuschem 11, no. 3 (2018): 527–531, 10.1002/cssc.201702405. 29316272

[40] R. Ciriminna , L. Albanese , F. Meneguzzo , and M. Pagliaro , “Hydrogen Peroxide: A Key Chemical for Today's Sustainable Development,” Chemsuschem 9, no. 24 (2016): 3374–3381, 10.1002/cssc.201600895. 27813285

[41] L. Li , Z. Hu , and J. C. Yu , “On‐Demand Synthesis of H_2_O_2_ by Water Oxidation for Sustainable Resource Production and Organic Pollutant Degradation,” Angewandte Chemie International Edition 59, no. 46 (2020): 20538–20544, 10.1002/anie.202008031. 32700466

[42] World Health Organization , Guidelines for Drinking‐Water Quality: Fourth Edition Incorporating the First and Second Addenda (WHO, 2022). 35417116

