# Supplementary material for: Polyelectrolyte Multilayer: Caged PTFE Particles in Water for Continuous, Sonochemical H2O2 Synthesis
Source: Adv Sci (Weinh). 2026 Aug 24:e77281. Online ahead of print. doi: 10.1002/advs.77281 (PMC13502278; doi:10.1002/advs.77281)
Supplement: Supplementary file 1 [file ADVS-9999-e77281-s001.pdf]

Supplementary information

Polyelectrolyte Multilayer – Caged PTFE Particles in Water for Continuous, Sonochemical H_2O_2 Synthesis

Rong Wang[1,2]‡, Bo Wang[1,2]‡, Tengyue Fu[2], Yue Yang[2], Chongling Cheng [1], Zhenhui Qi[1], Dayang Wang[2]**

1. State Key Laboratory of Digital Medical Engineering, School of Biological Science and Medical Engineering, Southeast University, Nanjing 210096, P. R. China

2. State Key Laboratory for Inorganic Synthesis and Preparative Chemistry, College of Chemistry, Jilin University, 130012, Changchun, China

* Corresponding authors' Email: chengchongling@seu.edu.cn (C. Cheng) and wangdayang@jlu.edu.cn (D. Wang)

‡ contribution equal to this work

1. Experimental Section……………………………………………………………S3

1.1 Materials

1.2 Methods

1.3 Characterization

2. Supporting information…………………………………………………………S8

2.1 Structural characterization and dispersion behavior of YSP and CSP particles (Fig. S1-S3)

2.2 Quantification of H_2O_2 generation and regulation of dissolved oxygen concentration (Fig. S4-S5)

2.3 Preservation of accessible hydrophobic PTFE interfaces within YSP architecture (Fig. S6-S7)

2.4 Proposed interfacial charge-transfer pathways and H_2O_2 formation mechanism

2.5 Structural dependence of YSP-enabled H_2O_2 generation (Fig. S8-S11)

2.6 General applicability of YSP architecture with different hydrophobic polymer cores (Fig. S12-S13)

2.7 Structural stability of YSP particles under prolonged ultrasonic irradiation (Fig. S14-S15)

2.8 Benchmarking of YSP-enabled H_2O_2 generation (Table S1)

2.9 Environmental applications enabled by in situ H_2O_2 generation (Fig. S16-S17, Table S2-S3)

2.10 Design and configuration of the YSP-assisted H_2O_2 generator (Fig. S18).

3. References………………………………………………………………….….S26

1. Experimental Section

1.1 Materials

Polyallylamine hydrochloride (PAH, Mw 120,000-200,000), sodium polystyrene sulfonate (PSS, Mw ~70,000), NaCl, Rhodamine 6G (R_6G^+, log P = 6.5, [S1]), sodium fluorescein (SF^-, log P = -0.08, [S2]), and acid orange 7(AO7) were purchased from Sigma-Aldrich (Shanghai, China). Absolute ethanol (>99%), hydrochloric acid, and NaOH were purchased from China Sinopharm Chemical Reagent Co., Ltd. Organophosphorus pesticides(dipterex), organophosphorus pesticide testing kits (Changsha weishi biotechnology Co., Ltd., China), and H_2O_2 testing paper (Jingwei biotechnology Co., Ltd., China) were purchased from a supermarket. Hydrophobic particles of PE, PTFE, PVC, and PP with mean particle sizes ranging from 0.1 to 30 μm were purchased from Dongguan Shuangfu Plastic Raw Materials Co., Ltd. All chemicals were used as received without further purification. Water with a resistivity of 18 $M\Omega \cdot cm^{-1}$ was prepared using a Millipore Milli-Q system and used in all experiments.

1.2 Methods

1.2.1 Preparation of degassed polyelectrolyte aqueous solutions

Aqueous solutions of polyelectrolytes PAH and PSS were prepared at a concentration of 1 $mg \cdot mL^{-1}$ in a 1 M NaCl solution, respectively. The pH of the PAH solution is adjusted to 3, 9, and 12 by the addition of 0.1 M NaOH.

To remove dissolved gases from these solutions, a freeze-pump-thaw process was repeated five times, following the procedure outlined in our previous work [S3]. In each cycle, the glass tube containing the polyelectrolyte solution was slowly immersed in liquid nitrogen to freeze the solution, preventing ice expansion. The tube was then evacuated for over 15 minutes to remove the gas from the headspace. Finally, the frozen solution was thawed at room temperature, allowing the dissolved gas to be drawn into the headspace. This process was repeated five times to ensure complete degassing.

1.2.2 The study of the surface coverage (f_{PEMs}) of PEM deposited on hydrophobic plastic particles substrates with the assistance of surface wettability

Since direct measurement of water contact angles on micron-sized particles is a colossal technical challenge, 0.1 g of the powder of bare or as-prepared PEM-coated hydrophobic particles were pressed under pressure of 2 MPa within 2 min to obtain thin plates. A 2 μL water droplet was placed on the film and imaged after 10 seconds to obtain the $\theta_{w/a}^{PEM}$ for pure or PEM-modified PTFE particles.

According to Cassie-Baxter equation [S4], the water wettability which reflected by the contact angles of water on as-prepared PEM modified hydrophobic PTFE particles surfaces in air ($\theta_{w/a}^{PEM}$) can be simply decomposed into the component of the hydrophilic PEMs surface and that of uncoated surfaces of the hydrophobic PTFE plates. Thus, the

apparent water contact angle ($\theta_{w/a}^{PEM}$) of PEM-PS in air can be descried by equation S1, S2:

$$\cos\theta_{w/a}^{PEM} = f_{PEM}\cos\theta_{w/a}^{PEM} + f_{PTFE}\cos\theta_{w/a}^{PTFE} \tag{S1}$$

$$f_{PEM} + f_{PTFE} = 1 \tag{S2}$$

where $\theta_{w/a}^{PEM}$ and $\theta_{w/a}^{PTFE}$ are the values of water-in-air contact angles of hydrophilic polar surface domains and hydrophobic non-polar surface domains, respectively. According to literatures [S3], the values of $\theta_{w/a}^{PEM}$ and $\theta_{w/a}^{PTFE}$ can be safely assumed to about 7.5° and 120°, respectively. The f_{PEM} and f_{PTFE} are the surface coverage of the PEM-PTFE modified surface and that of the uncoated surfaces of the hydrophobic PP plates, respectively.

Using the same method, the proportion of CF_2 functional groups on the surfaces of PTFE particles with varying sizes was determined. Based on literature references [S3], the water-air contact angles $\theta_{w/a}^{CF_2}$ and $\theta_{w/a}^{CH_2}$ can be reliably assumed to be approximately 120° and 100°, respectively. Subsequently, the coverage of CF_2 groups on the solid surface can be calculated using the relevant equations.

1.2.3 The study of H_2O_2~~H_2O_2~~ generation under ultrasonication treatment

PTFE, CSP, and YSP particles were added to aqueous solutions, ensuring consistent PTFE content across all samples. The particle-dispersed solutions were placed in beakers and subjected to ultrasonic treatment in a bath where ice was added to maintain a stable external water temperature. After sonication, the particles were filtered from the solutions to yield clear, transparent liquid samples, and the H_2O_2 concentration ($C_{H_2O_2}$) in these samples was measured.

Similarly, PVC, PP, and PE particles, along with YSP particles prepared using these materials as cores, underwent the same procedure to determine the resulting H_2O_2 concentration ($C_{H_2O_2}$). To mitigate the influence of temperature variations caused by sonication on H_2O_2 production, ultrasonic treatment was paused for 1 h after every 1-hour session, allowing the water temperature to stabilize before resumption.

The amount of H_2O_2 generated in the solution were measured according to the following process. After filtered the particles, the concentration of H_2O_2 in the collected solution was detected by an iodometric method. Specifically, 0.4 M potassium iodide (50.0 μL) and 0.1 mM ammonium molybdate tetrahydrate (50.0 μL) were first added into the collected solution (3.0 mL). Then, these solutions were fully mixed and placed for 3 min. Finally, the color solution was detected by UV-vis spectrophotometry at 352 nm. The standard curve and equation of UV-vis absorbance were shown in Fig. S5 in supporting information.

1.2.4

Control of dissolved oxygen concentration and its determination by the iodometric method

To investigate the effect of dissolved oxygen on sonochemical H_2O_2~~H_2O_2~~ production, the dissolved O_2 concentration (C_{O_2}) in the aqueous reaction system was adjusted by bubbling mixtures of high-purity O_2 and Ar at different flow-rate ratios into the water before and during ultrasonic irradiation. All experiments were conducted in an open glass vessel at atmospheric pressure (1 atm) and room temperature (25 °C). Before ultrasonication, the actual dissolved O_2 concentration in the aqueous dispersion was quantitatively determined using the standard iodometric titration method.

The iodine method, standardized by ISO 5813-1983, is a precise technique for measuring dissolved oxygen concentrations in water samples. It's effective for a wide range, from 0.2 mg·L^{-1} up to around 20 mg·L^{-1}. To ensure accurate results, it's crucial to prevent bubble formation during the procedure. Typically, equal volumes of manganese sulfate ($MnSO_4$, 2.2 M) and alkaline iodide solution (2.1 M potassium iodide (KI) and 8.7 M potassium hydroxide (KOH)) are added to the water sample (V_0) until the brown-orange manganese hydroxide ($Mn(OH)_3$) precipitates completely. Then, 2 M sulfuric acid (H_2SO_4) was added to dissolve the precipitate and release I_2 to reacts with I^- to form I_3^-. Finally, those triiodide ions are titrated with a standardized sodium thiosulfate ($Na_2S_2O_3$) solution ($C_{Na_2S_2O_3}$ =0.0015 mol/kg) with 1 mL starch solution (10 g/L) as the indicator. The titration continues until the blue color disappears, indicating the endpoint. Therefore, the concentration of dissolved oxygen (DO) can be calculated using the following equation:

$$C_{O_2} = (V_{Na_2S_2O_3} \times C_{Na_2S_2O_3}) / V_0 \quad \text{(S3)}$$

Where C_{O_2} is the dissolved oxygen concentration in mol·L^{-1}, $V_{Na_2S_2O_3}$ is the volume of $Na_2S_2O_3$ used in the titration (mL), $C_{Na_2S_2O_3}$ is the concentration of the $Na_2S_2O_3$ (mol /L) and V_0 is the volume of the water sample (mL). Each sample was tested 5 times in parallel and averaged to obtain an accurate value.

1.2.5 The study of the presence of pesticide on the vegetable with the help of pesticide residue detection kits

To detect pesticide residues on the surface of vegetables, pesticide residue detection kits were used in accordance with the national standard GB/T 5009.119-2003. The method for detecting organophosphorus pesticide residues on vegetable surfaces is as follows:

i) Sample preparation: The non-woven cloth was firstly used to remove any soil on the surface of the vegetables. Then, cut the vegetables into small pieces with a diameter of approximately 1 cm.

ii) Extraction: Weigh out 5g of the prepared vegetable pieces and immerse them in 10 mL of extraction solvent. Shake the mixture by hand 50 times, then let it

stand for 2 minutes.

iii) Test card preparation: Remove the protective film from the test card. Drop the supernatant liquid from step 2 onto the white circle on the test card and let it stand for more than 10 minutes.

iv) Color development: Fold the test card in half so that the red circle completely overlaps the white circle. Press firmly with your fingers for only 3 minutes.

v) Result interpretation: Observe any color change within the white circle on the test card. If there is no color change, it indicates no pesticide residue. A blue color change suggests the presence of pesticide residue.

Note: A blank control test using only the extraction solvent for each detection.

1.3 Characterization

1.3.1 Contact angle measurements were conducted using a drop shape analyzer – DSA100 system (KRüSS GmbH, Germany) at ambient temperature. The obtained plastic particles were compressed under 2 MPa for 2 min to obtained a tablet. Then, the water-in-air contact angles ($\theta_{w/a}$) on prepared tablet were measured by placing a water droplet of 2 μL atop the surfaces.

1.3.2 The absorption/transmittance spectra were obtained in the wavelength range of 250 – 800 nm using a Shimadzu UV-2700 UV-vis spectrometer (Japan).

1.3.3 The confocal fluorescence microscopic images were obtained using Nikon C2 confocal fluorescence microscopes.

1.3.4 The surface morphology of particles and their cross-ponding element mapping were characterized by means of field emission scanning electron microscope (FE-SEM, SU4800, Hitachi, Japan).

1.3.5 Raman spectroscopy was carried out on a Horiba LabRAM HR evolution instrument in the Raman shift range of 200-1800 cm^{-1} under irradiation of 532 nm (50 mW), respectively, and the accumulation time of 60 s was applied to collect all the spectroscopic data**.**

1.3.6 The X-ray photoelectron spectroscopy (XPS) measurements have been conducted on a Thermo Fisher Scientific K-Alpha, in a vacuum of 1×10^{-9} mBar, using an Alka ray source (hv = 1486.6 eV), the working voltage is 15 kV and the filament current is 10 mA. The signal accumulation was performed for five to ten cycles. The pass energy is set at 30 eV.

1.3.7 LC-MS analysis was performed using an Ultimate 3000 UHPLC coupled with a

Q Exactive mass spectrometer (Thermo Scientific, US). Samples were filtered through a 0.22 μm microporous membrane before direct injection. The instrument operated in positive ion mode with the HESI ion source voltage set to 3.8 kV, covering a full scan range of 100 – 1000 m/z at a resolution of 70,000 FWHM. Chromatographic separation was achieved on a Waters Atlantis T3 column (100 mm × 3.0 mm, 3.0 μm) maintained at 40 °C, using methanol and 0.1% formic acid aqueous solution as the mobile phase at a flow rate of 0.4 mL/min.

1.3.8 FTIR analysis were conducted on a Thermo scientific Nicolet iS50 on a range from 400 to 3000 cm^{-1}.

1.3.9 Electron paramagnetic resonance (EPR) were recorded on a JES-FA 200 EPR spectrometer with an X-band regime (~9.4 GHz) in the magnetic field range of 297 ~ 377 mT at room temperature. The 5,5-dimethyl-1-pyrroline N-oxide (DMPO) as a spin-trapping agent. The EPR spectra of ultrsonicgenerated •OH radicals were recorded in the aqueous suspensions and those of ultrsonicgenerated $•O_2^-$ radicals in the methanol suspesions.

2. Support information

2.1 Structural characterization and dispersion behavior of YSP and CSP particles

Fig. S1 Chemical structures of polyallylamine hydrochloride (PAH) with pKa value of 8.7 [S5] and sodium polystyrene sulfonate (PSS).

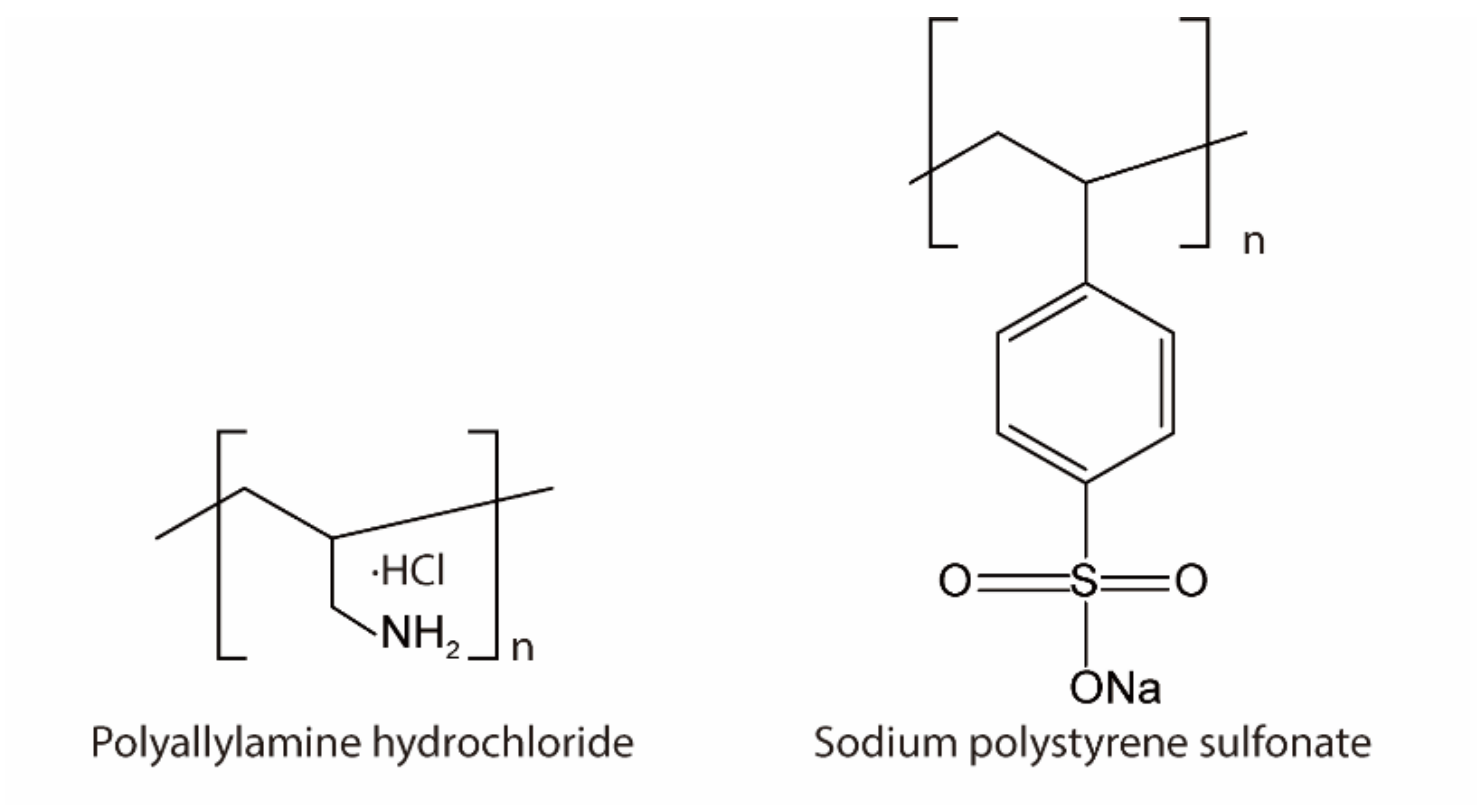

Fig. S2 Plots of PEM surface coverage proportion (f_{PEM}) on hydrophobic PTFE particles towards the number of deposition cycles (m) through recycle immersing the aqueous solution of PAH at pH 3 (black squares), 12 (red circles), and 9 (blue triangles) and which calculated based on the water contact angle($\theta_{w/a}^{PEM}$) and Cassie equation (S1). The results indicate that complete PEM coverage of the PTFE particle surface is achieved after 10 cycles of LbL deposition using PAH solution at pH 9.

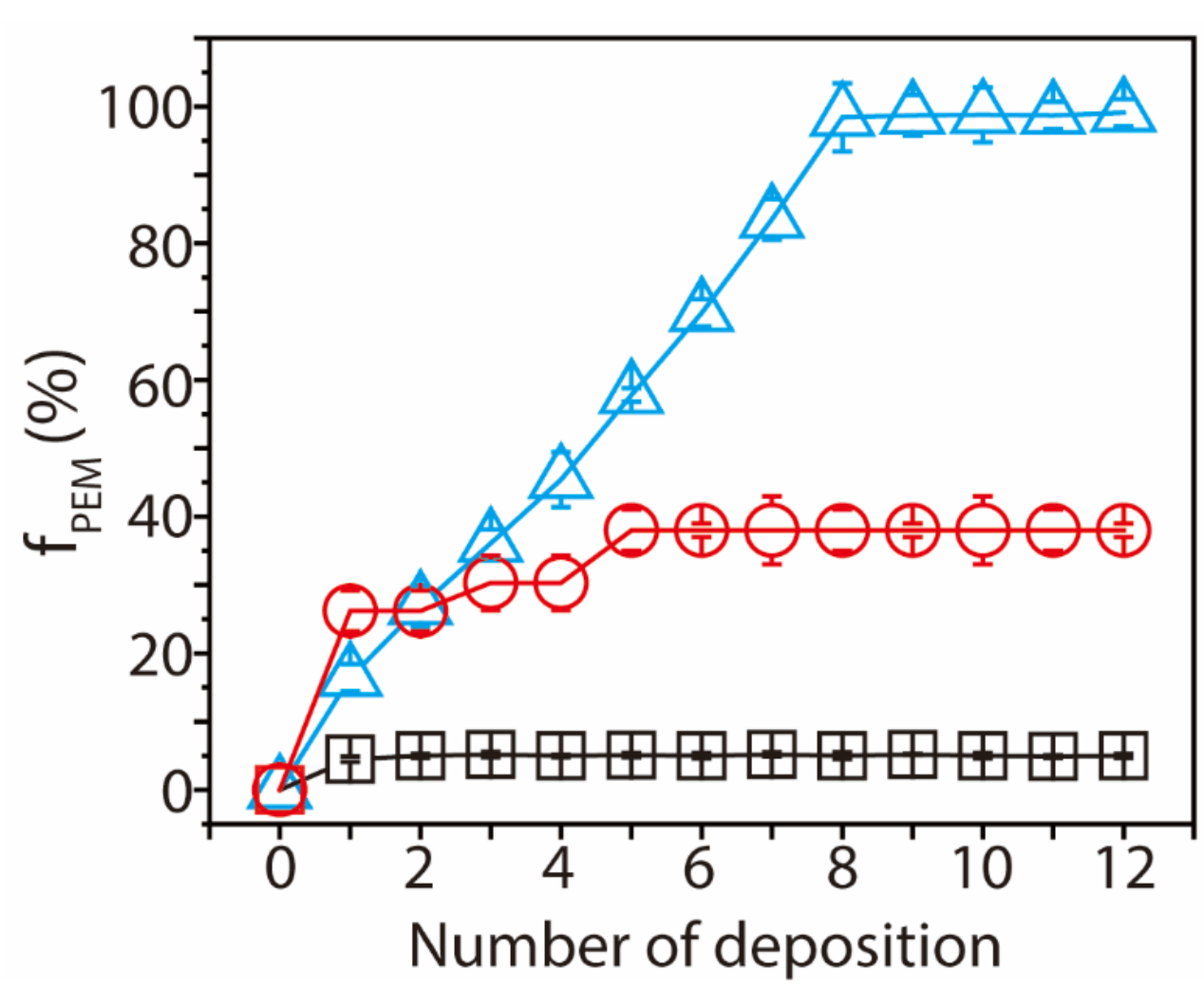

Fig. S3 Sunlight images (left panel) and Tyndall Effect observations (right panel) of aqueous solutions containing **a)** PTFE particles partially coated with PEM. The experimental results demonstrate that insufficient PEM coverage on PTFE particles leads to only partial dispersion in the aqueous medium, preventing complete and stable dispersion.

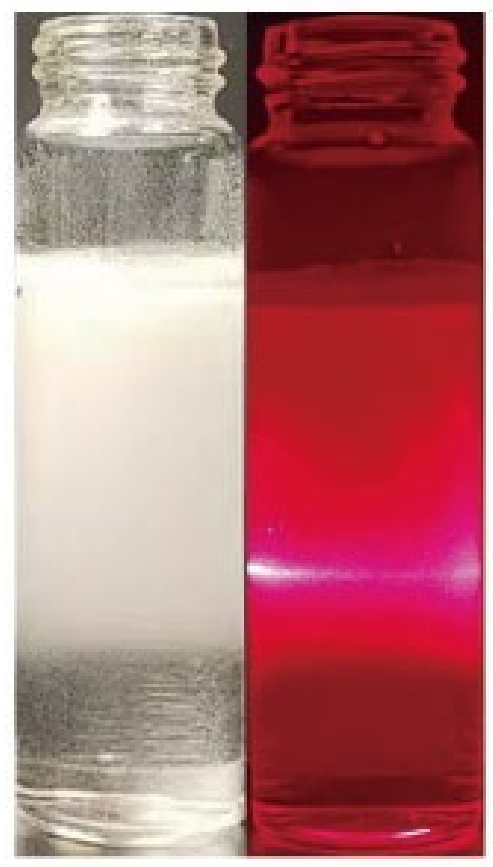

2.2 Quantification of H_2O_2~~H_2O_2~~ generation and regulation of dissolved oxygen concentration

Fig. S4 a) UV-Vis absorption spectra of potassium iodide/ammonium molybdate tetrahydrate (volume ratio = 1:1) aqueous solution after reaction with water containing different concentrations of H_2O_2. **b)** Plot of absorbance intensity (I) at 352 nm of the potassium iodide/ammonium molybdate tetrahydrate/H_2O_2 mixed solution as a function of H_2O_2 concentration ($C_{H_2O_2}$). A linear relationship is observed, enabling the quantification of H_2O_2 concentration in aqueous solutions through absorbance measurements.

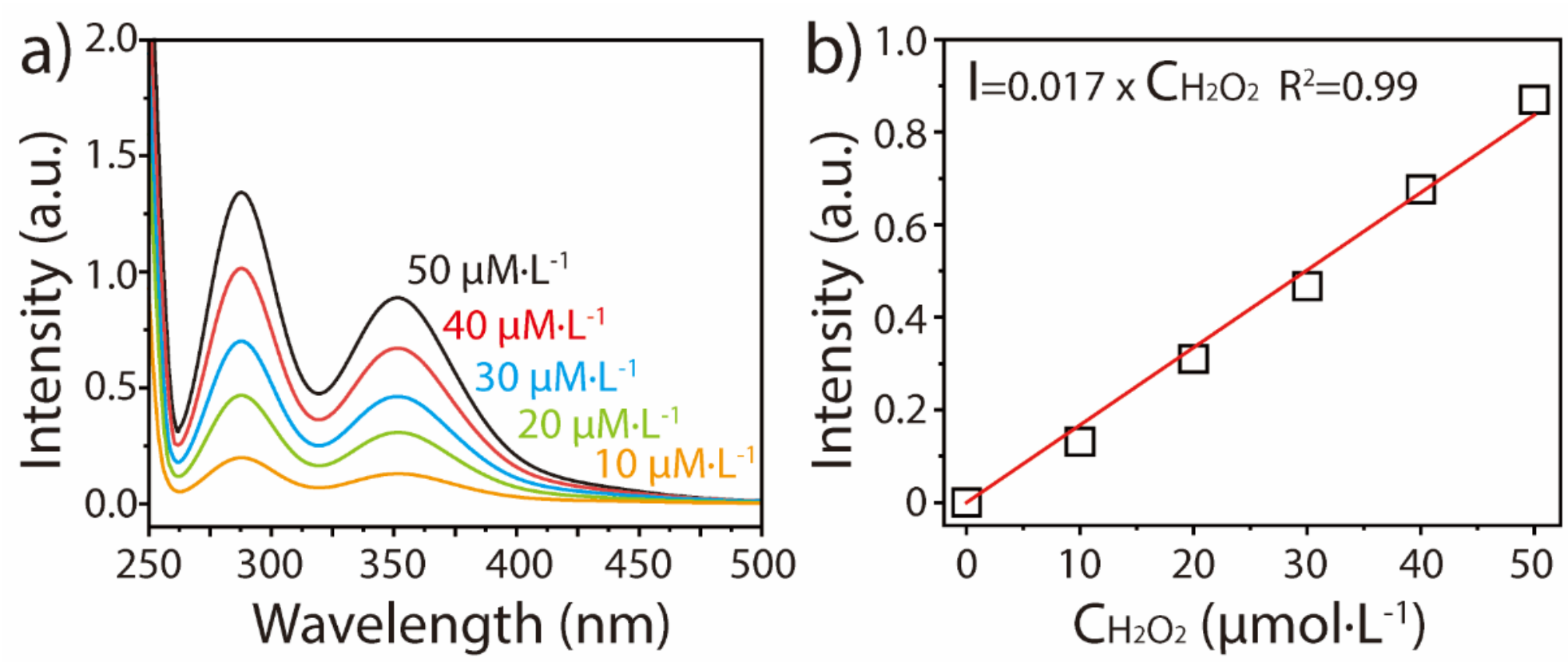

Fig. S5 Control and measurement of dissolved O_2 concentrations. (a) Time-dependent dissolved O_2 concentrations during with the same O_2/Ar gas mixture O_2 bubbling in initially degassed water (black squares) and untreated water (red circles). (**b**) EquilibriumStabilized dissolved O_2 concentrations obtained after bubbling water with using O_2/Ar mixtures containing 0-50 vol% O_2 for 60 min. Black squares represent the theoretical dissolved O_2 concentrations calculated according to Henry's law, whereas red circles represent the experimentally measured values. Dissolved O_2 concentrations was determined by the iodometric method at 25 °C and 1 atm. Data are presented as mean ± standard deviation (n = 3).

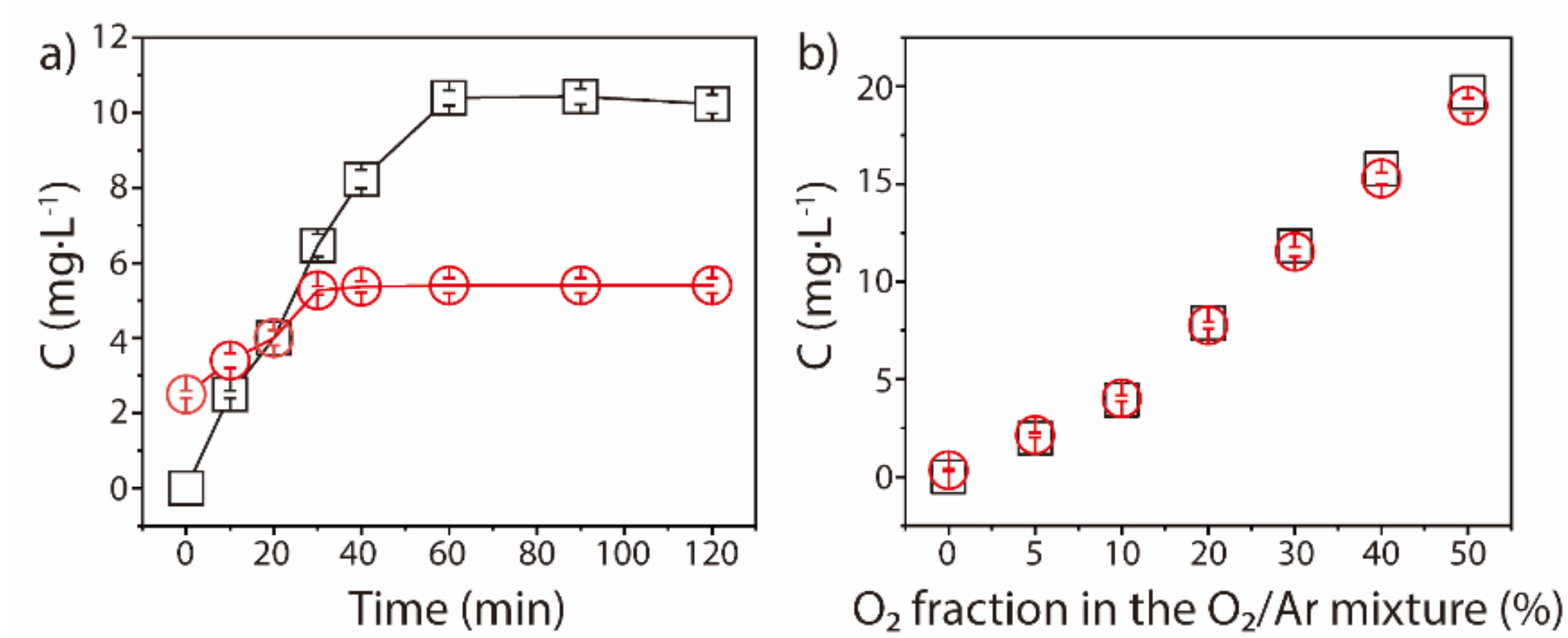

2.3 Preservation of accessible hydrophobic PTFE interfaces within YSP architecture

Fig. S6 UV-Vis transmittance spectra (400-800 nm) of the as-prepared aqueous dispersions of YSP particles before (black line) and after (blue line) introducing oxygen under sonication, followed by manual shaking to redisperse the particles (red line). The results confirm that the dispersed YSP particles completely floated to the surface after oxygen introduction and sonication, facilitating easy collection of the PTFE particles from the aqueous phase.

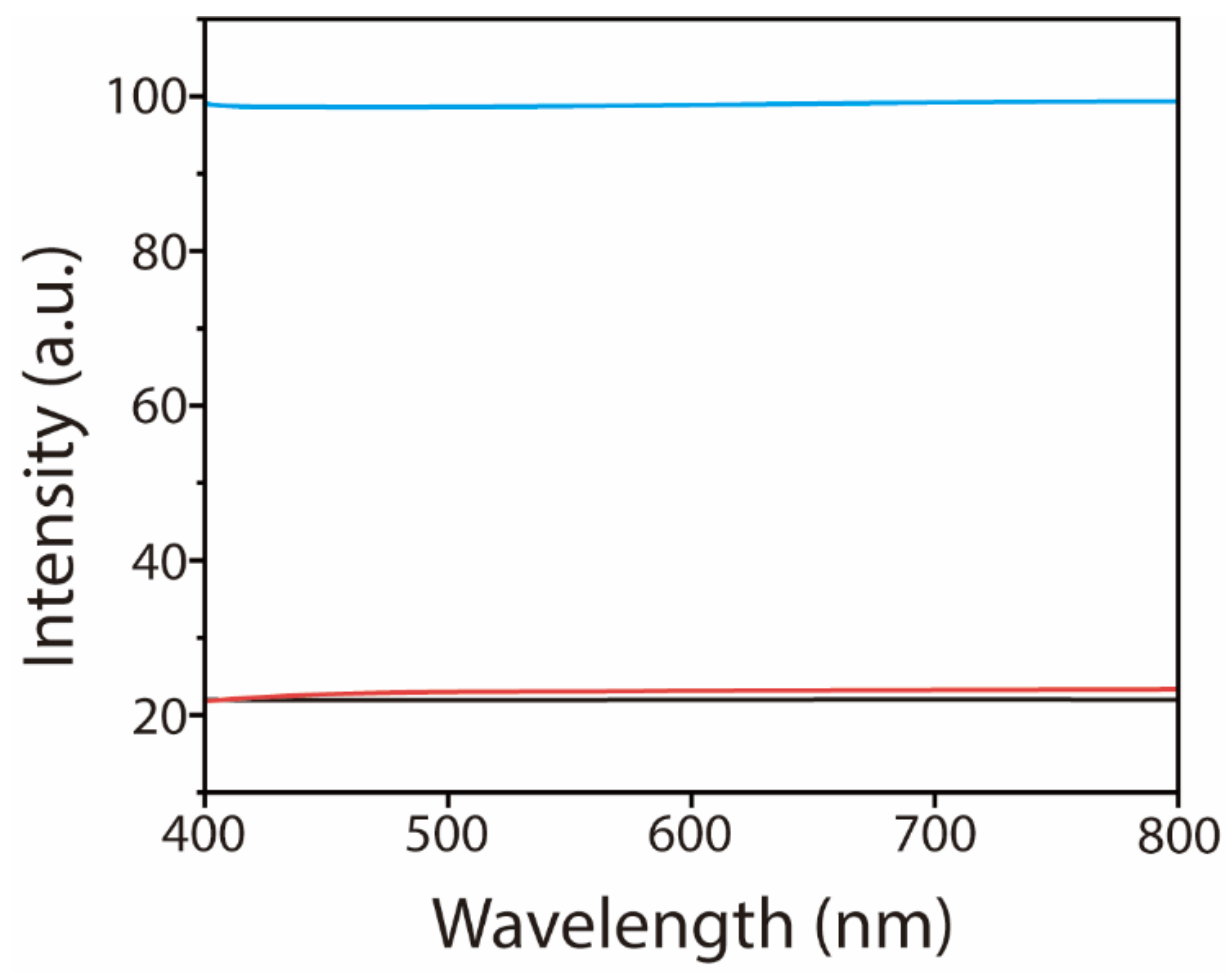

Fig. S7 Sunlight images (left panel) and Tyndall Effect observations (right panel) of aqueous solutions of CSP after introducing oxygen under sonication treatment for 1 h. Unlike YSP particles, CSP particles did not float significantly in the aqueous solution, as their hydrophobic surfaces were fully covered by the hydrophilic PEM.

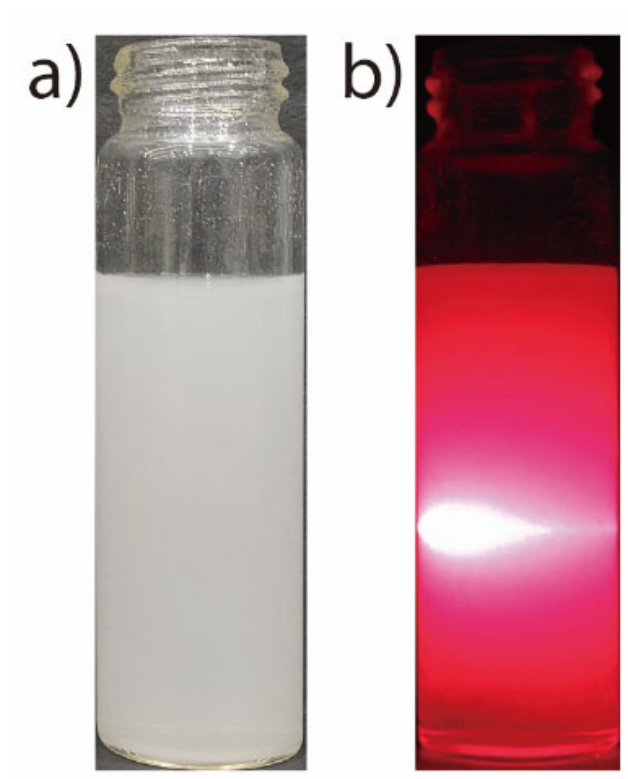

2.~~5~~ 4 Proposed interfacial charge-transfer pathways and H_2O_2 ~~H_2O_2~~ formation mechanism.

The internal void of the yolk–shell particles provide a confined interfacial region between the hydrophobic PTFE yolk and the hydrophilic polyelectrolyte shell. Water can penetrate through the hydrated shell and directly contact the PTFE surface, while the hydrophobic PTFE interface and the surrounding void favor the retention of pre-existing gas nuclei and heterogeneous cavitation nucleation. Under the applied ultrasound field, these gas nuclei undergo repeated oscillation, growth, and collapse, thereby promoting localized acoustic cavitation near the PTFE–water interface.

The collapse of cavitation bubbles generates localized high temperature and pressure, shock waves, and high-velocity microjets. These effects intensify solid–liquid collisions and repeated contact–separation at the PTFE–water interface, thereby promoting high-frequency solid–liquid contact electrification (SL-CE). Therefore, the internal void of the YSPs does not directly generate cavitation but provides a favorable microenvironment for gas-nucleus retention and heterogeneous cavitation nucleation. Meanwhile, the hydrophilic polyelectrolyte shell improves particle dispersion in water while maintaining the accessibility of the hydrophobic PTFE surface.

Owing to the strong electron-withdrawing ability of the fluorinated groups in PTFE, cavitation-enhanced contact electrification facilitates electron transfer from adjacent water molecules to the PTFE surface. The resulting interfacial charges subsequently initiate coupled water oxidation and oxygen reduction pathways for H_2O_2 generation.

2.~~5~~4.1 Water Oxidation Reaction Pathway

During SL-CE, electrons are transferred from interfacial water molecules to the PTFE surface, producing water radical cations:

$$\mathrm{PTFE} + \mathrm{H_2O} \xrightarrow{\mathrm{SL-CE}} \mathrm{PTFE}^*(\mathrm{e}^-) + \mathrm{H_2O}^{\cdot+} \qquad (\text{~~S3~~S4})$$

The resulting water radical cations rapidly react with neighboring water molecules to generate hydroxyl radicals:

$$\mathrm{H_2O}^{\cdot+} + \mathrm{H_2O} \rightarrow HO^{\bullet} + \mathrm{H_3O}^+ \qquad \text{~~)~~} \quad (\text{S5})$$

2.~~5~~4.2 Oxygen Reduction Reaction Pathway

Meanwhile, electrons accumulated on the negatively charged PTFE surface, denoted as $\mathrm{PTFE}^*(\mathrm{e}^-)$, are transferred to dissolved oxygen, generating superoxide radicals through the oxygen reduction reaction:

$$\mathrm{PTFE}^*(\mathrm{e}^-) + \mathrm{O_2} \rightarrow \mathrm{PTFE} + \mathrm{O_2^{\cdot-}} \qquad (\text{S~~5~~6})$$

Acoustic cavitation continuously promotes interfacial collisions, contact electrification, charge transfer, and mass transport, thereby sustaining this oxygen-dependent reduction pathway.

2.~~5~~4.3 Subsequent H_2O_2 Formation Pathways

2.~~5~~4.3.1 Dominant Pathway: Protonation and Disproportionation of $O_2^{\bullet-}$

The generated $O_2^{\bullet-}$ intermediates act as key precursors and undergo protonation to form hydroperoxyl radicals:

$$O_2^{\bullet-} + H_3O^+ \rightarrow HO_2^{\bullet} + H_2O \qquad (S7)$$

The hydroperoxyl radicals subsequently undergo disproportionation to produce H_2O_2:

$$2HO_2^{\cdot} \rightarrow H_2O_2 + O_2 \qquad (S8)$$

2.~~5~~4.3.2 Secondary Pathway: Self-Recombination of $HO^{\bullet}$

Hydroxyl radicals may also directly recombine to form H_2O_2:

$$2HO^{\bullet} \rightarrow H_2O_2 \qquad (S9)$$

However, this pathway provides only a minor contribution under the present experimental conditions.

2.~~5~~4.4 Mechanistic Interpretation

Experiments conducted under controlled dissolved-oxygen conditions showed that negligible H_2O_2 accumulated in deaerated water, indicating that direct $HO^{\bullet}$ recombination alone was insufficient to produce detectable H_2O_2. In contrast, increasing the dissolved-O_2 concentration markedly enhanced the $O_2^{\bullet-}$ EPR response and H_2O_2 yield. These results demonstrate that dissolved O_2 acts as an essential electron acceptor and that the $O_2^{\bullet-}$-mediated pathway described in Equations S6–S8 is the dominant route for ultrasound-driven H_2O_2 production.

The enhanced H_2O_2 production of the YSPs can therefore be attributed to the structural coupling between acoustic cavitation and interfacial contact electrification. The hydrophilic polyelectrolyte shell improves the aqueous dispersion of the particles, whereas the internal void preserves the accessibility of the hydrophobic PTFE surface and provides a confined region favorable for gas-nucleus retention and heterogeneous cavitation nucleation. The oscillation and collapse of interfacial cavitation bubbles generate microjets and intense local solid–liquid collisions, thereby enhancing contact electrification and electron transfer at the PTFE–water interface. The transferred electrons are subsequently captured by dissolved O_2 to form $O_2^{\bullet-}$, which undergoes protonation and disproportionation to produce H_2O_2. To further clarify the structural factors governing this process, the effects of PTFE yolk size and surface chemistry were systematically investigated.

2.~~6~~ 5 Structural dependence of YSP-enabled H_2O_2~~H_2O_2~~ generation~~Influence of PTFE core size and surface properties on H_2O_2 generation~~

Fig. ~~S9~~ S8 Plots of the generated H_2O_2 concentration in pristine water media with varying concentrations of YSP particles towards sonication time. The core plastic particle diameters in the YSP particles were 0.1 µm **(a)**, 0.2 µm **(b)**, 1 µm **(c)**, 5 µm **(d)**, 10 µm **(e)**, and 30 µm **(f)**. These results highlight the impact of particle size on H_2O_2 production efficiency under sonication conditions.

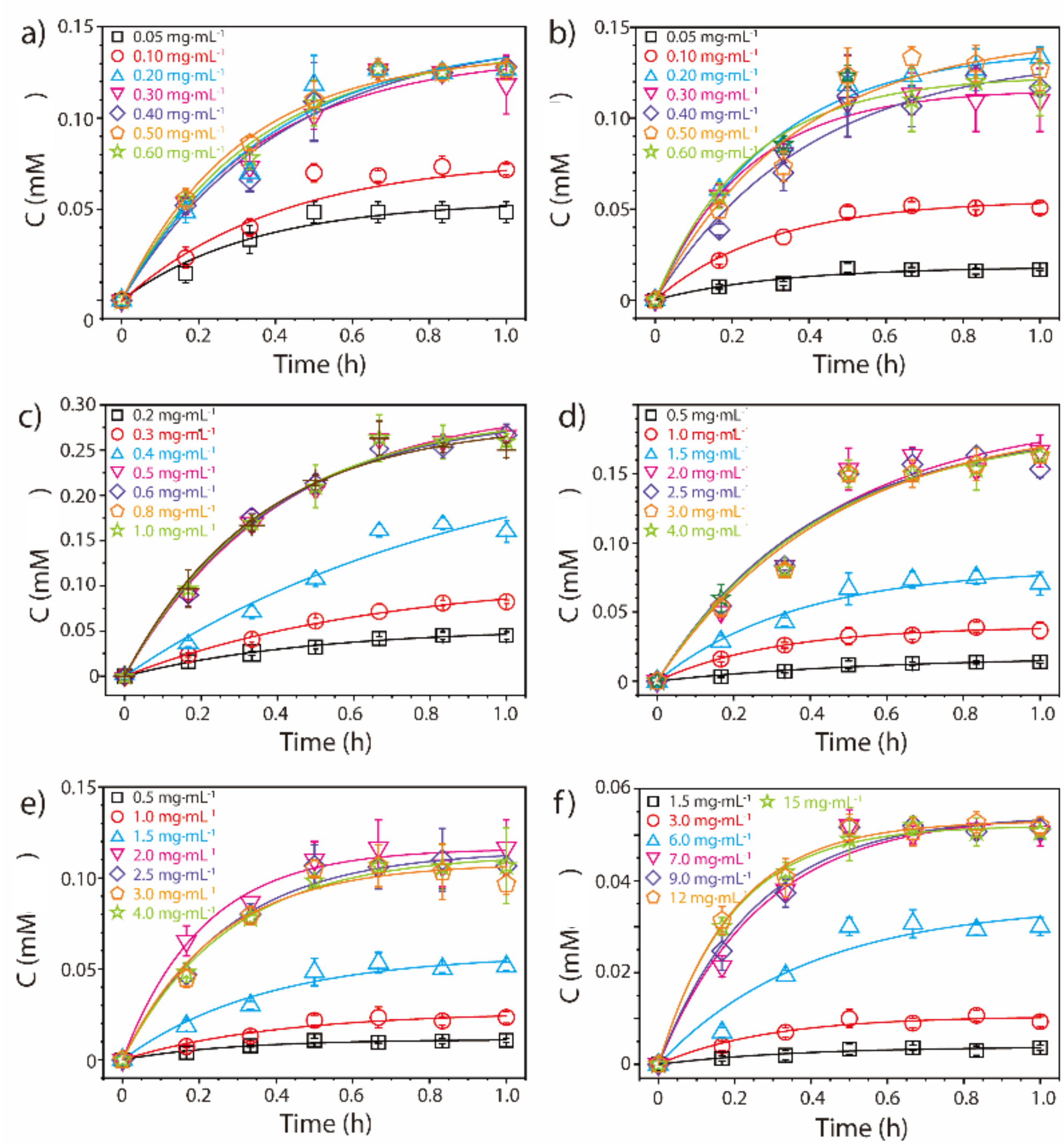

Fig ~~**S10**~~ **S9** Plots of generated ~~H_2O_2~~H_2O_2 concentration in aqeous media (left) and normalized H_2O_2 generation molecular number rate per unit surface area of YSP particles (right) towards YSP particle concentration. The core plastic PTFE particle diameters in the YSP particles were 0.1 µm **(a)**, 0.2 µm **(b)**, 5 µm **(c)**, 10 µm **(d)**, and 30 µm **(e)**. These data illustrate the influence of particle size and concentration on H_2O_2 production efficiency, providing insights for optimizing YSP-based catalytic systems.

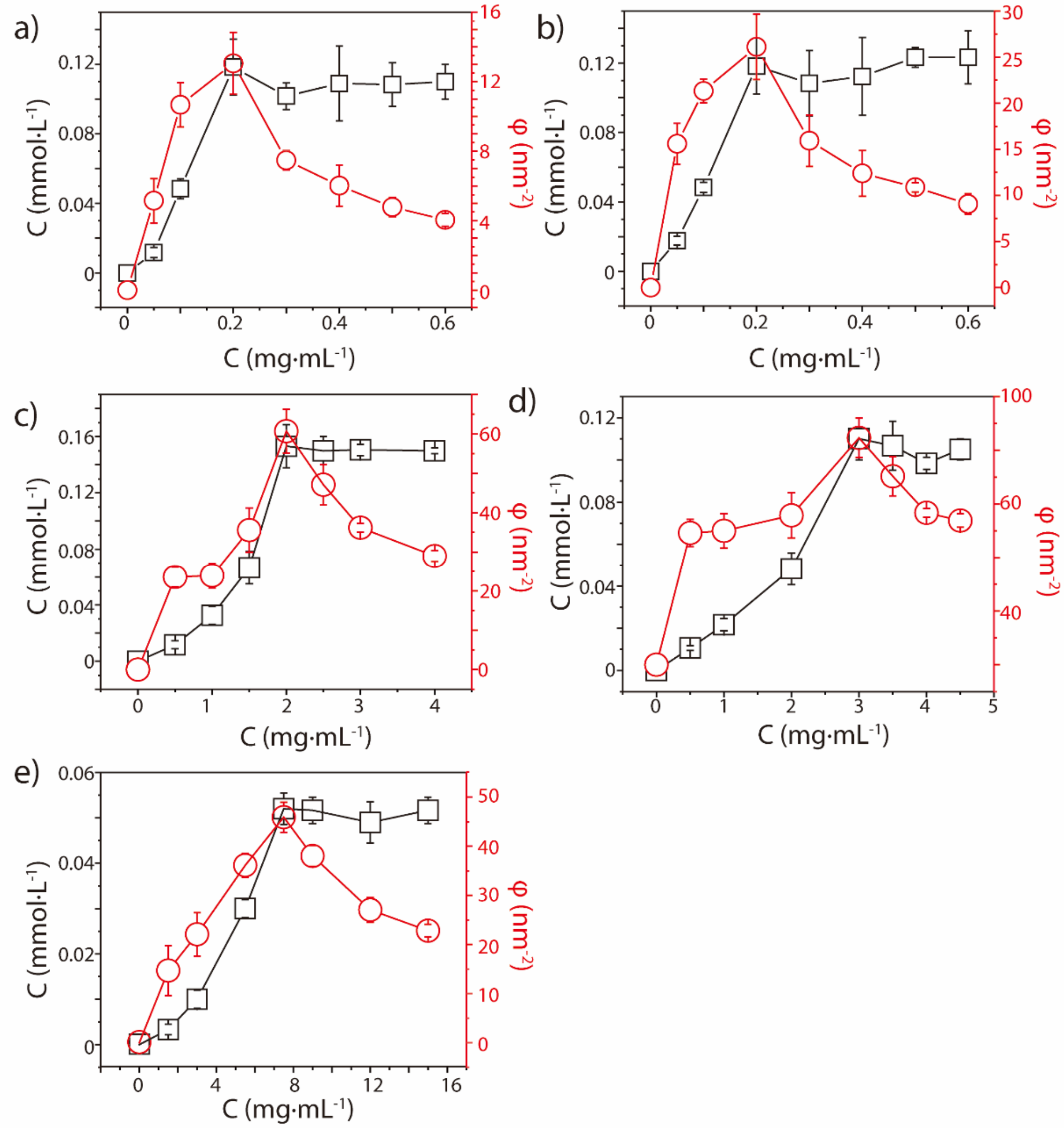

Fig. ~~S11~~ S10 Images of 2 μL water droplets on thin table plates prepared from PTFE particles of varying sizes, with corresponding contact angles on the surface of PTFE indicated below each image. The results highlight the effect of particle size on the hydrophobic properties of the surface of particles as reflected in the contact angles.

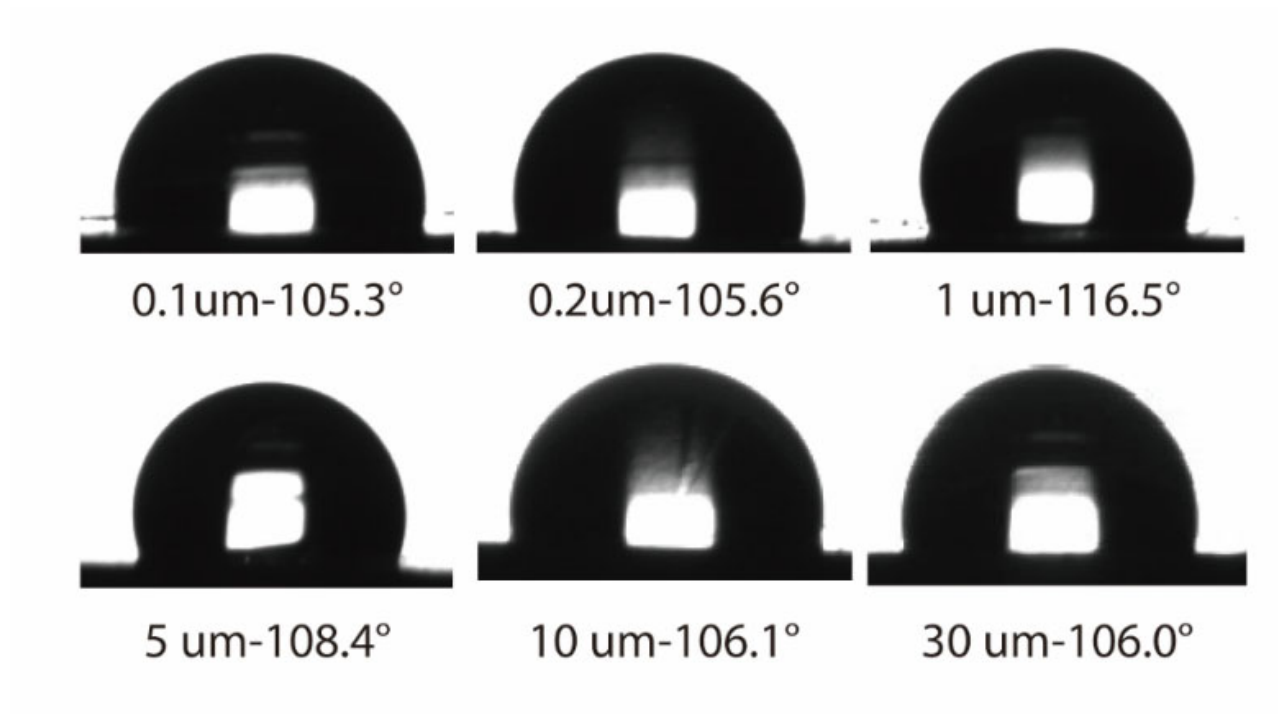

Fig. ~~S12~~ S11 High-esolution XPS Spectra of Carbon for PTFE Particles with Varying Diameters: 0.1 µm **(a)**, 0.5 µm **(b)**, 1 µm **(c)**, 5 µm **(d)**, 10 µm **(e)**, and 30 µm **(f)**. The proportion of CF_2 functional groups on the PTFE particle surface, as determined from XPS analysis, aligns closely with the results obtained through contact angle measurements, demonstrating consistency between the two methods in characterizing surface functionalization.

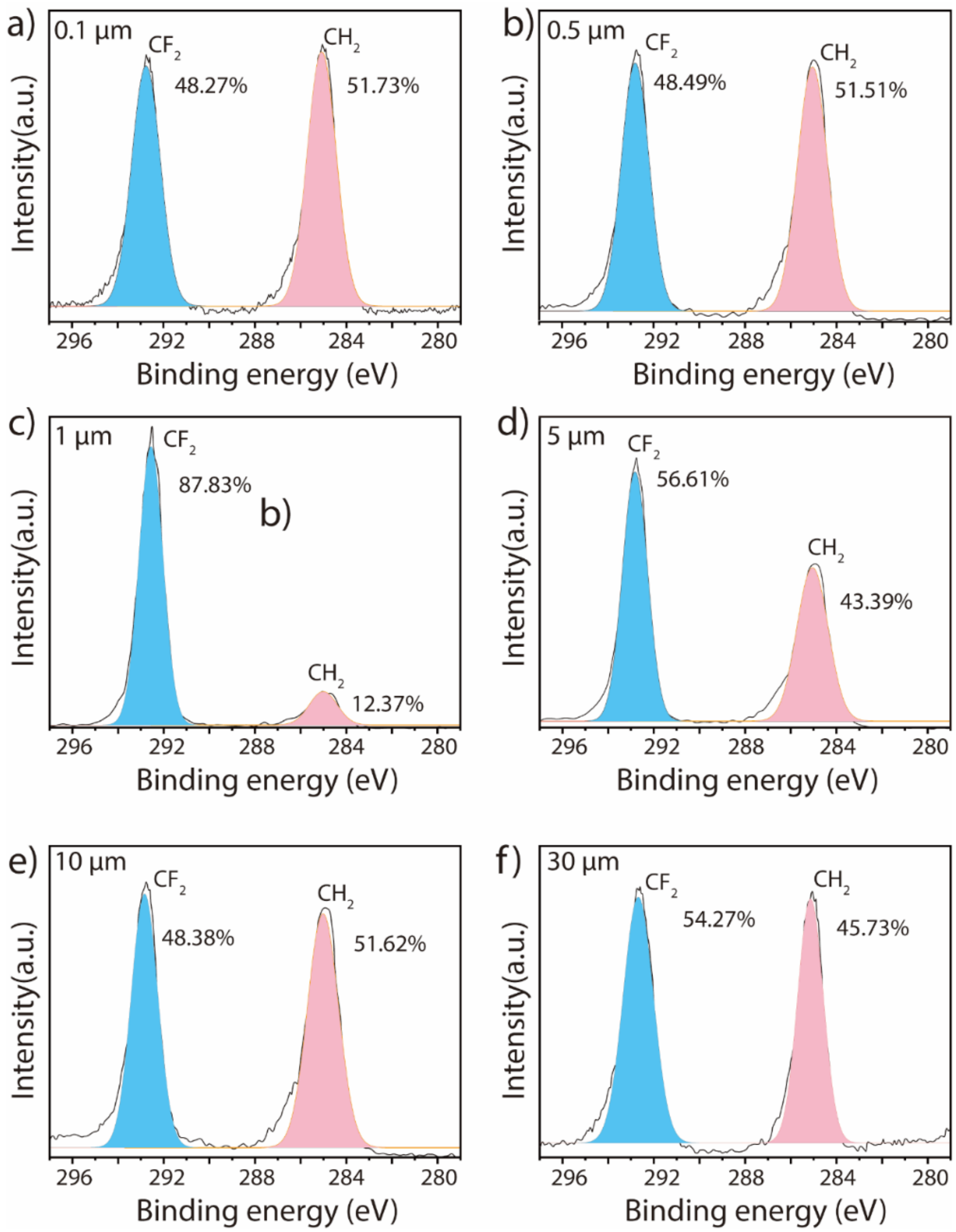

2.76 General applicability of YSP architecture with different hydrophobic polymer cores

Fig. S132- Plots of generated H_2O_2 concentration by YSP with PE **a)**, PP **b)**, and PVC **c)** cores in deionized water towards the reaction time under sonciation treatment.

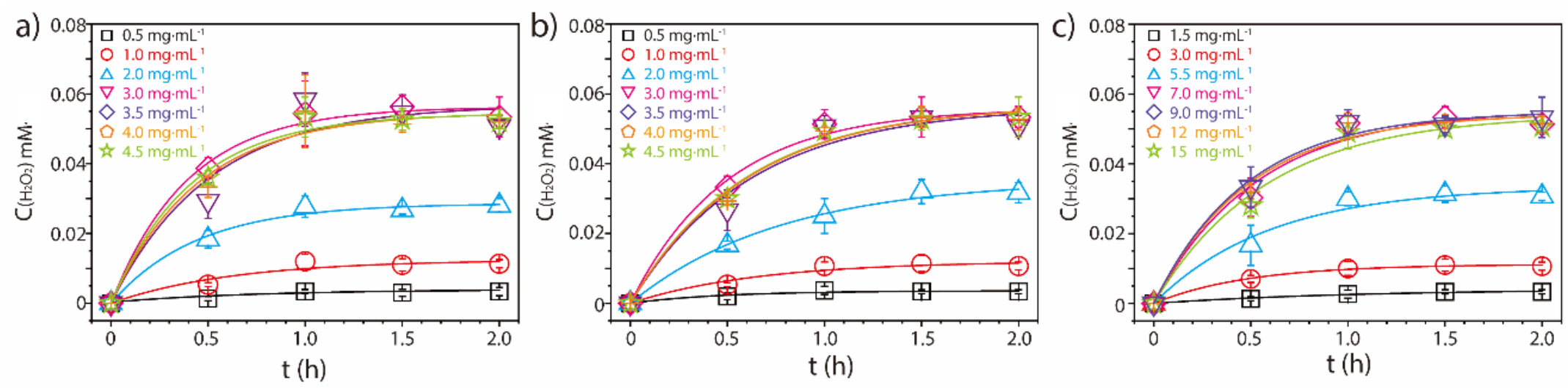

Fig. S14 S13 Plots of generated $H_2O_2$$H_2O_2$ concentration in pristine water media (left) and normalized $H_2O_2$$H_2O_2$ generation rate per unit surface area of YSP particles (right) towards YSP particle concentration under sonciation treatment. The core plastic particle were PE **a)**, PP **b)**, and PVC **c)**.

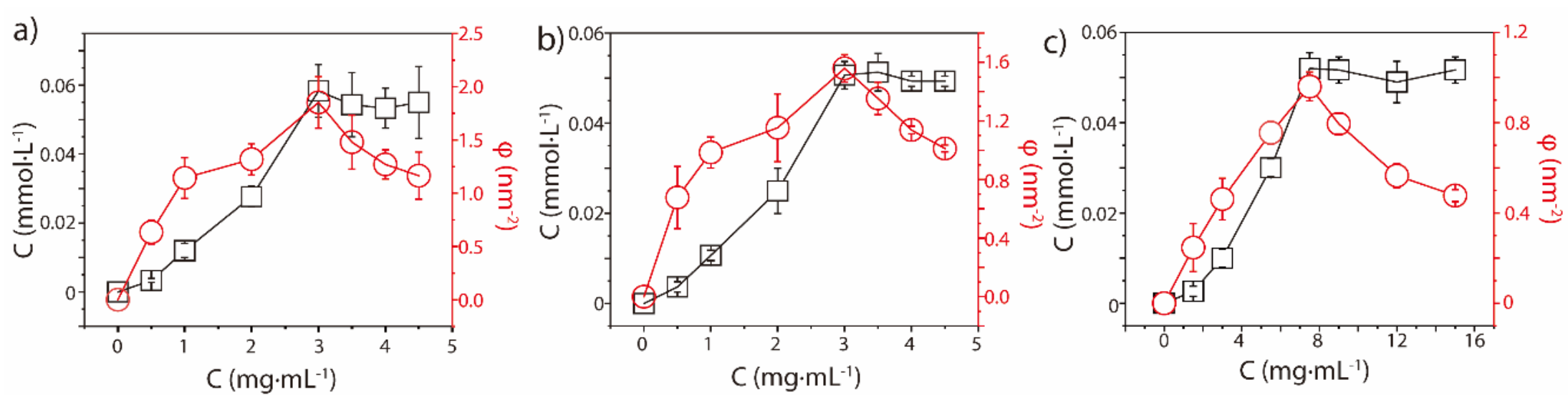

2.7 Structural stability of YSP particles under prolonged ultrasonic irradiation

Fig. ~~S15~~ S14 The SEM images of the YSP particles before **(a)** and after **(b)** sonication for 12 hours. The morphology of the YSP particles remained unchanged, demonstrating that the shape of the particles is stable during the sonication process.

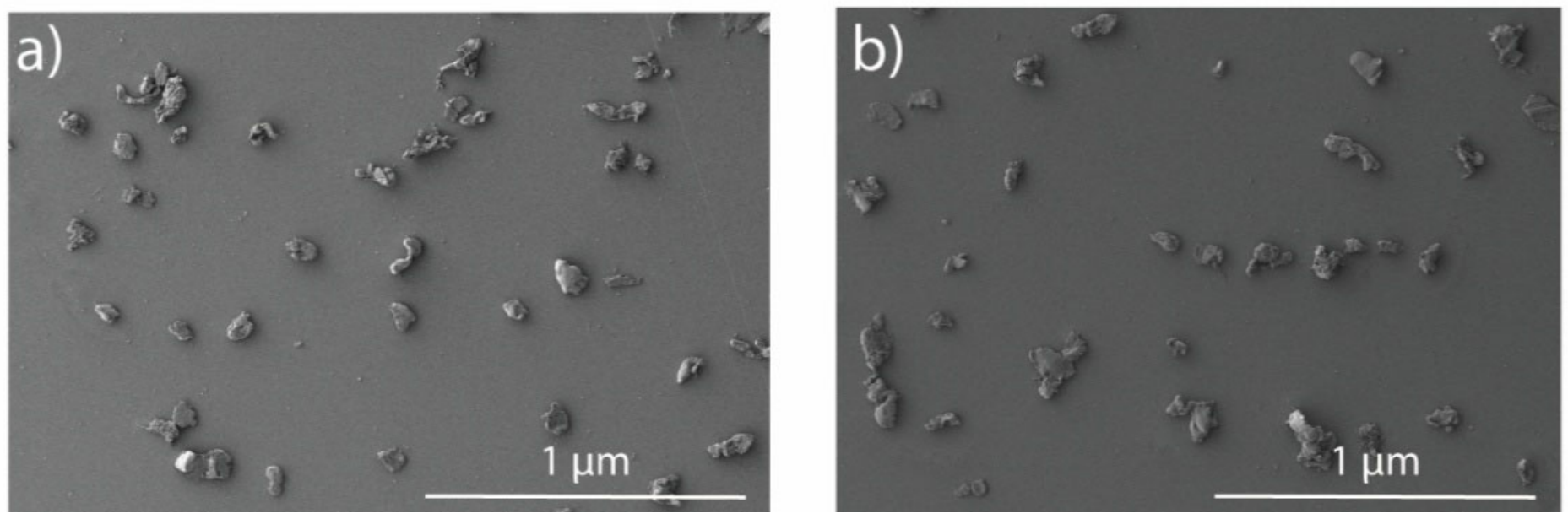

Fig. S15 The surface XPS spectra of the YSP particles before **a)** and after **b)** ultrasonica treatment within 12 h. The smilar spectra confirm the sonicaition treatment has little infuence on the surface properties of YSP particles.

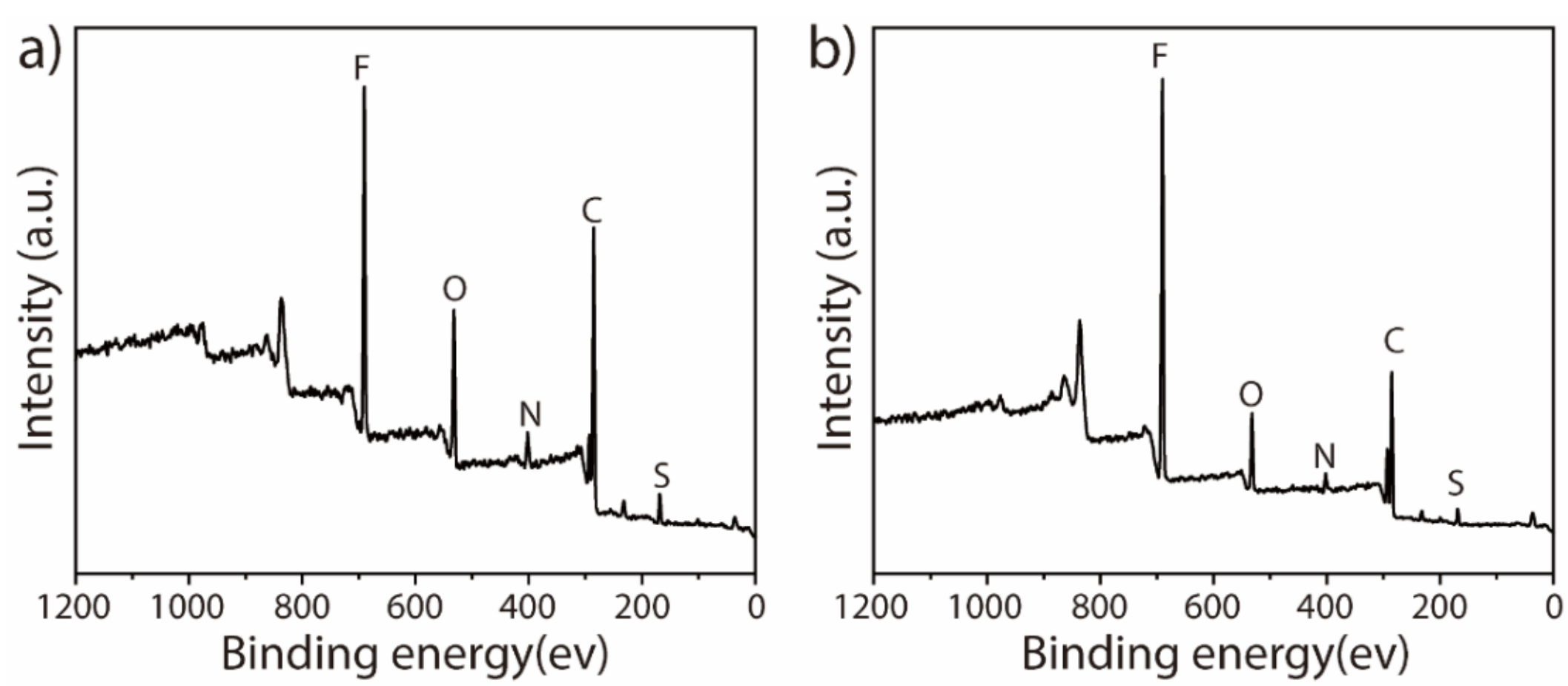

2.8 Benchmarking of YSP-enabled H_2O_2~~H_2O_2~~ generation

Table S1. Comparison of reported H_2O_2~~H_2O_2~~ generation systems without sacrificial agents..~~Comparison of currently available photocatalysts for photocatalytic generation of H_2O_2 in the absence of sacrificial agents.~~

	Catalysts	Reaction solutions	H_2O_2 formation rate (μmol g^{-1} h^{-1})	References
0	YSP	Pure water	3340	This work
1	PAH-cored COFs	Pure water	3820	[S7]
2	TACOF-1-COOH	Pure water	3542	[S8]
3	PAQ-TABPB	Pure water	3351	[S9]
4	Bpt-CTF	Pure water	3268.1	[S10]
5	sulfur-deficient $ZnIn_2S_4$ nanosheets onto UiO-66-NH_2	Pure water	3200	[S11]
6	B-ZnInS	Pure water	3121	[S12]
7	EO-COF	Pure water	2675	[S13]
8	HCOF	Pure water	2113.9	[S14]
9	P doped $ZnIn_2S_4$ catalyst	Pure water	2107.66	[S15]
10	PCN	Pure water	2063.21	[S16]
11	Through-space charge transfer complexes based on terpyridine Bi-functionalization	Pure water	2063	[S17]
12	CuX-dptz	Pure water	1874	[S18]
13	COFs with high-density hydrazone linkages	Pure water	1588	[S19]
14	TP-BT-0F-COF	Pure water	1435.8	[S20]
15	benzotrithiophene-based covalent organic frameworks with spatially separated redox centers	Pure water	1407	[S21]
16	COF-TPB	Pure water	1205	[S22]
17	S1-Zn	Pure water	1163	[S23]
18	Ag FECC	Pure water	1075.5	[S24]
19	BON-M	Pure water	970.27	[S25]
20	fl-CN-530	Pure water	952	[S26]
21	donor-acceptor COFs	Pure water	605	[S27]
22	BiOCl	Pure water	560	[S28]
23	Ag-CTC1	Pure water	229.6	[S29]
24	Au/$BiVO_4$	Pure water	172.2	[S30]
25	$Co_{14}(L-CH_3)_{24}$	Pure water	146.6	[S31]
26	MOC-AuNP/g-C_3N_4	Pure water	120.21	[S32]
27	ZnPPC-NBCN	Pure water	114	[S33]
28	Ag/t$BaTiO_3$	Pure water	80.3	[S34]
29	COF-O	Pure water	15	[S35]

2.9 Environmental applications enabled by in situ $H_2O_2$$H_2O_2$ generation

Fig. S17 (a) UV-Vis spectra of aqueous solutions of AO7 at concentrations ranging from 0.05 to 10 mg L^{-1}, and **(b)** plot of absorbance intensity at the 485 nm peak versus AO7 concentration. The results indicate that the detection limit of AO7 via UV-Vis spectroscopy is 50 μmol L^{-1}. When AO7 was degraded to undetectable levels using H_2O_2 generated by the YPS catalyst, its concentration in the aqueous phase was confirmed to be below 50 μg L^{-1} (50 ppb), meeting the World Health Organization's standards for general organic pollutants in potable water. [S36]

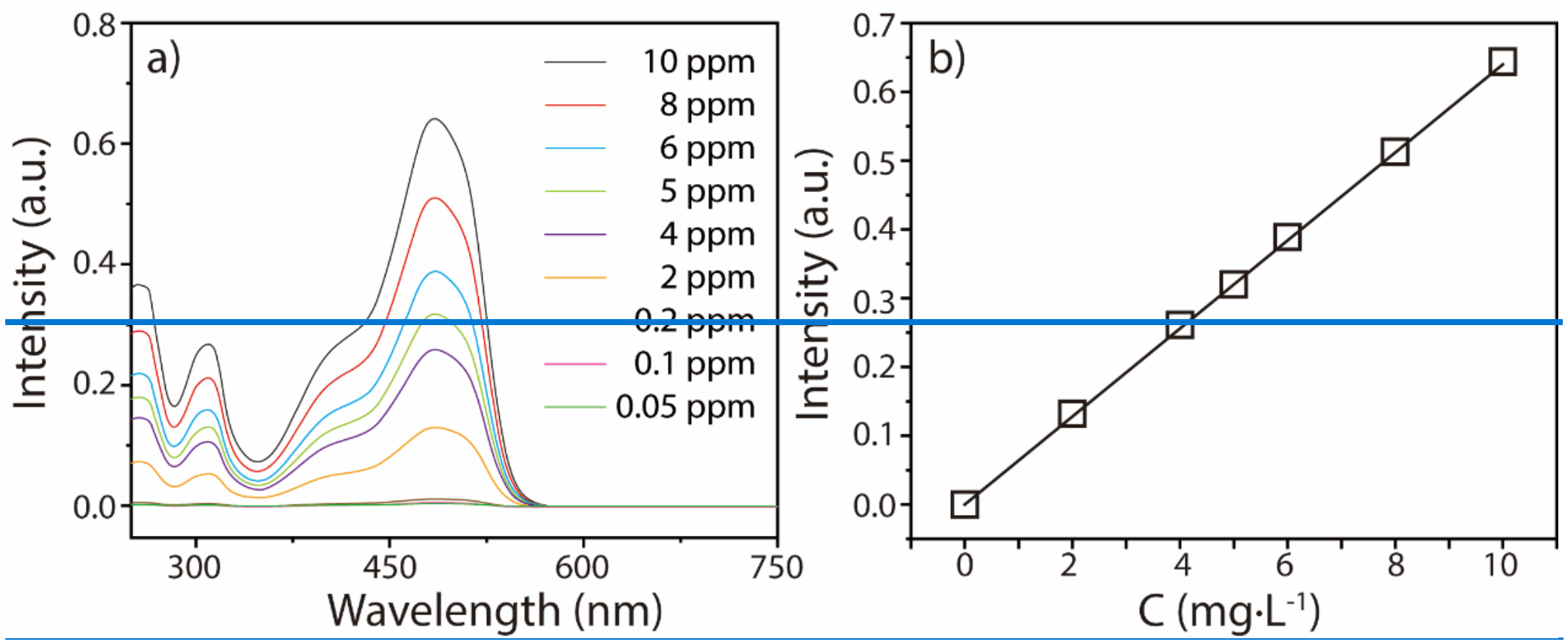

Fig. -S16 UV-Vis spectra of AO7 before and after adsorption by 10 mg of YSP particles incubated for varying durations. The experimental results demonstrate that simple adsorption alone is insufficient to remove AO7 from the aqueous phase.

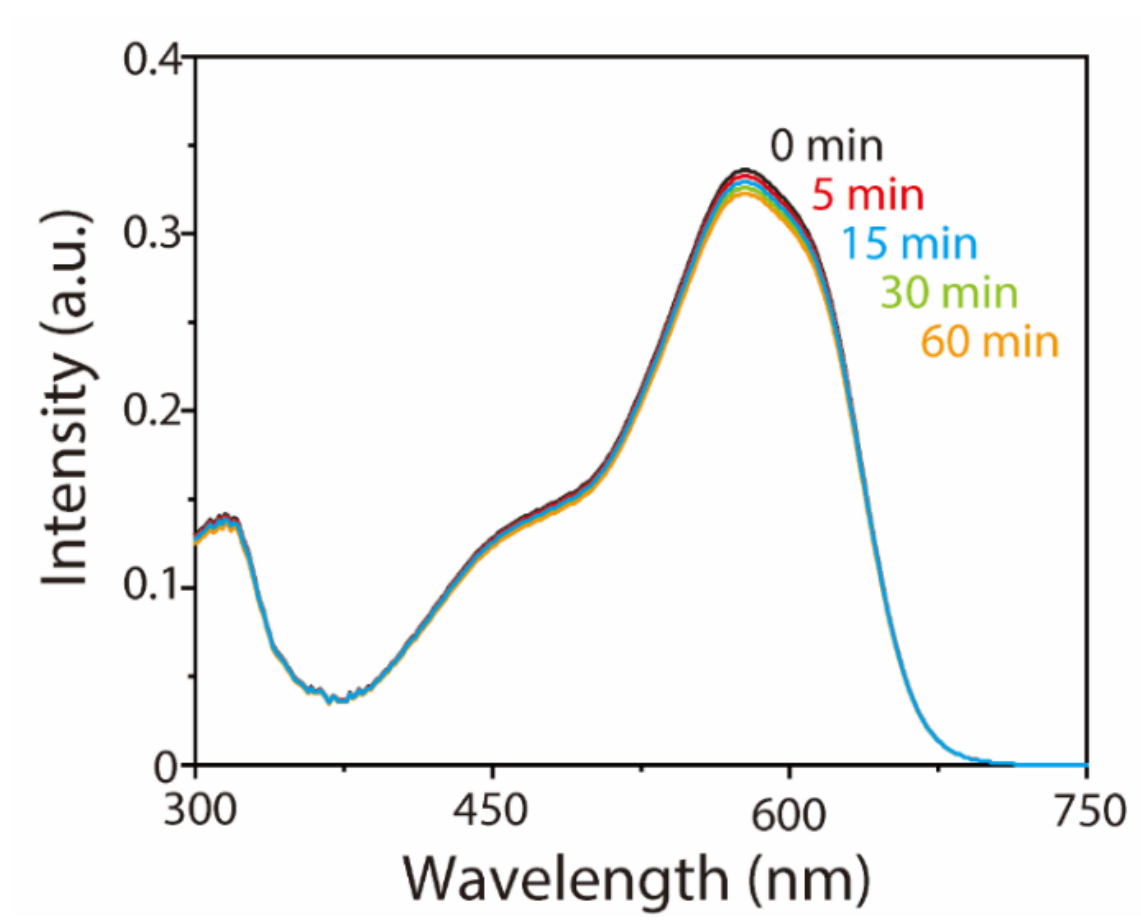

Fig. S17 (a) UV-Vis spectra of aqueous solutions of AO7 at concentrations ranging from 0.05 to 10 mg L^{-1}, and **(b)** plot of absorbance intensity at the 485 nm peak versus AO7 concentration. The results indicate that the detection limit of AO7 via UV-Vis spectroscopy is 50 μmol L^{-1}. When AO7 was degraded to undetectable levels using H_2O_2 generated by the YSP catalyst, its concentration in the aqueous phase was confirmed to be below 50 μg L^{-1} (50 ppb), meeting the World Health Organization's standards for general organic pollutants in potable water. [S36]

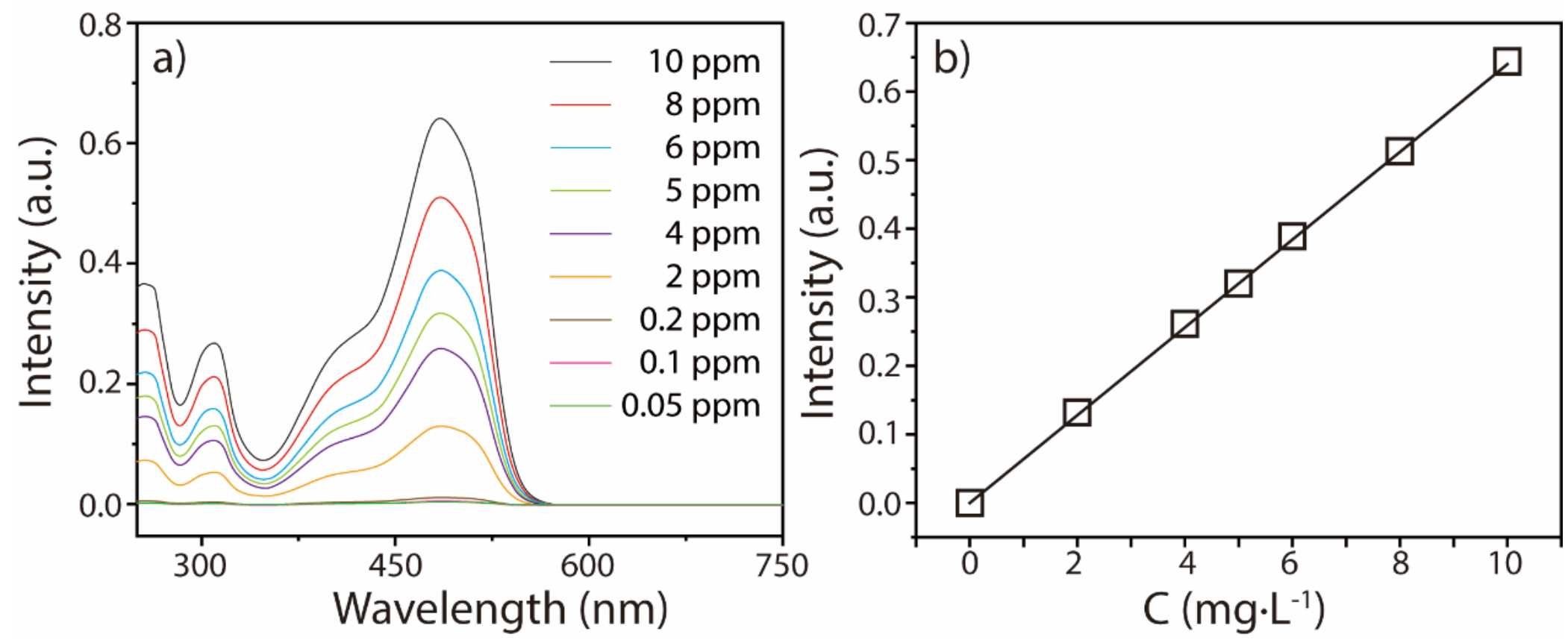
a)
10 ppm
8 ppm
6 ppm
5 ppm
4 ppm
2 ppm
0.2 ppm
0.1 ppm
0.05 ppm
Intensity (a.u.)
Wavelength (nm)
0.0
0.2
0.4
0.6
0.8
300
450
600
750
b)
Intensity (a.u.)
C (mg·L-1)
0.0
0.1
0.2
0.3
0.4
0.5
0.6
0.7
0
2
4
6
8
10

Table S2 Chemical structures of AO7 and its ultrasonic H_2O_2 degradation products, as well as their mass-to-charge ratios (m/z) identified by mass spectrometry (ESI-MS).[S37]

m/z	327	338	299	273	173
**Chemical structure**	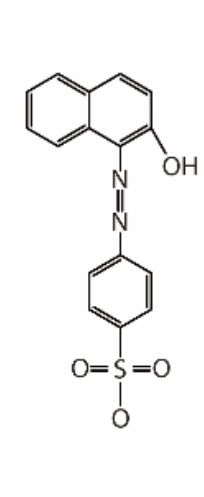	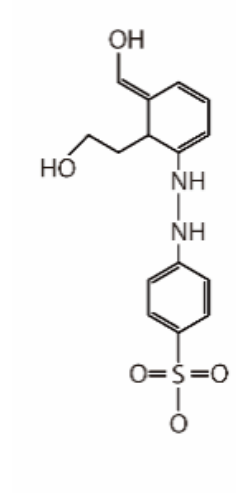	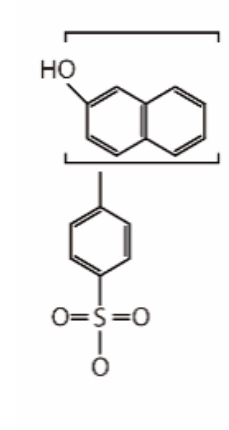	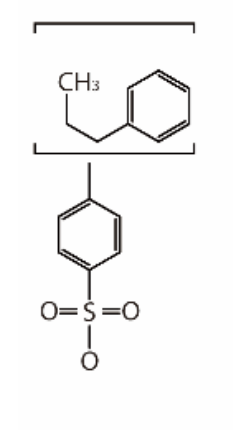	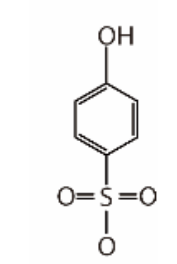

Table S3 Chemical structures of the organophosphorus compound (Dichlorvos) and its degradation products formed via ultrasonic with aqueous solution of YSP particles with concentration of 1 mg·mL^{-1}, along with their mass-to-charge ratios (m/z) identified by mass spectrometry (ESI-MS).

m/z	256	221	185	109	93
**Chemical structure**	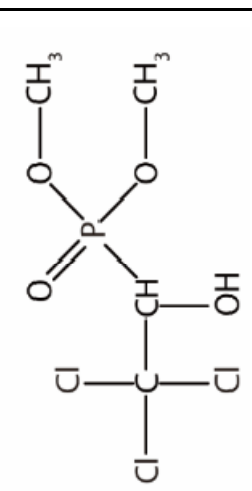	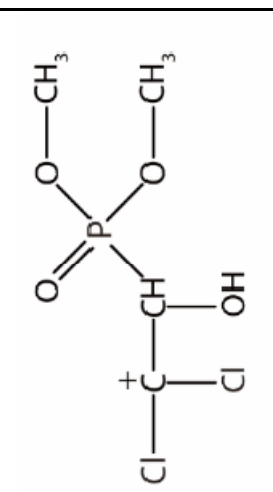	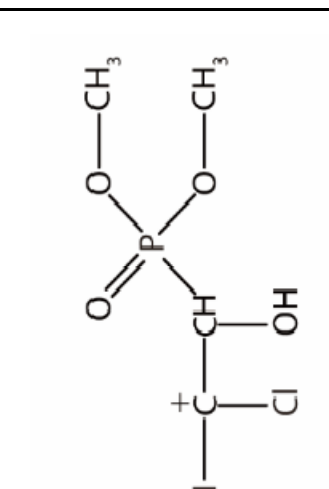	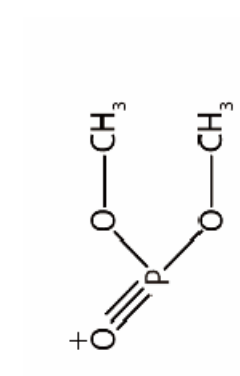	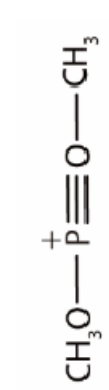

2.110 Design and configuration of the YSP-assisted $H_2O_2$$H_2O_2$ generator

Fig. S198. External appearance and internal configuration of the home-built YSP-facilitated H_2O_2 generator. The device has overall dimensions of 130 × 80 × 70 mm³ and operates at an ultrasonic frequency of 60 kHz with a nominal power of 100 W. The internal assembly comprises a 90 mm ultrasonic probe, a plastic bag containing the YSP dispersion, and a 40 mm oxygenator. The YSP-filled plastic bag was positioned between the ultrasonic probe and the oxygenator to enable simultaneous ultrasonic irradiation and oxygen supply during H_2O_2 generation.

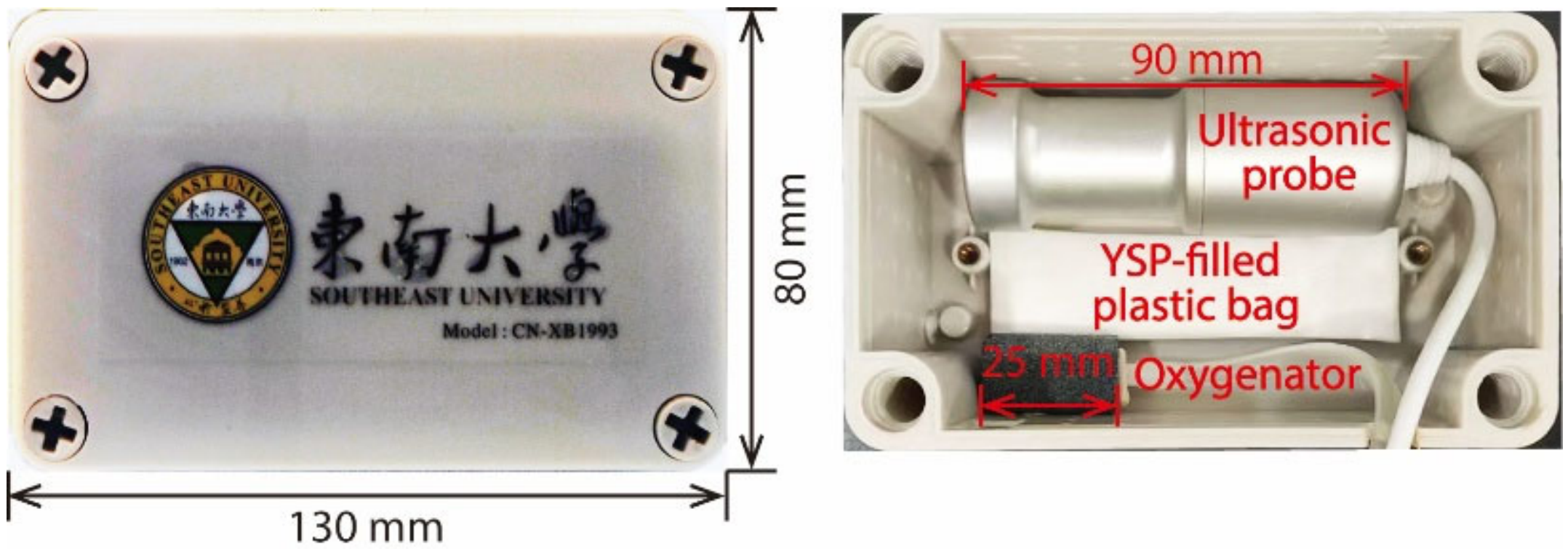
東南大學
SOUTHEAST UNIVERSITY
Model : CN-XB1993
80 mm
130 mm
90 mm
Ultrasonic probe
YSP-filled plastic bag
25 mm
Oxygenator

3. References

[S1] L. F. Mottram, S. Forbes, B. D. Ackley, and B. R. Peterson, "Hydrophobic Analogues of Rhodamine B and Rhodamine 101: Potent Fluorescent Probes of Mitochondria in Living *C. elegans*," *Beilstein Journal of Organic Chemistry* 8 (2012): 2156–2165.

[S2] Y. Oba and S. R. Poulson, "Octanol-Water Partition Coefficients (*K*ow) vs. pH for Fluorescent Dye Tracers (Fluorescein, Eosin Y), and Implications for Hydrologic Tracer Tests," *Geochemical Journal* 46, no. 6 (2012): 517–520.

[S3] B. Wang, T. Fu, C. Cheng, and D. Wang, "Layer-by-Layer Assembly of Polyelectrolytes on Hydrophobic Particles in Aqueous Milieu for Efficient Dye Removal," *Chemical Communications* 59 (2023): 12479–12482.

[S4] A. B. D. Cassie, "Contact Angles," *Discussions of the Faraday Society* 3 (1948): 11–16.

[S5] D. Hong and S. H. Yang, "Cationic Polymers for Coating Living Cells," *Macromolecular Research* 26 (2018): 1185–1192.

[S6] F. Caruso, H. Lichtenfeld, E. Donath, and H. Möhwald, "Investigation of Electrostatic Interactions in Polyelectrolyte Multilayer Films: Binding of Anionic Fluorescent Probes to Layers Assembled onto Colloids," *Macromolecules* 32, no. 7 (1999): 2317–2328.

[S7] X. Chi, Z. Zhang, M. Li, Y. Jiao, X. Li, F. Meng, B. Xue, D. Wu, and F. Zhang, "Vinylene-Linking of Polycyclic Aromatic Hydrocarbons to π-Extended Two-Dimensional Covalent Organic Framework Photocatalyst for H_2O_2 Synthesis," *Angewandte Chemie International Edition* 64 (2025): e202418895.

[S8] H. Xu, Y. Wang, Y. Xu, Q. Wang, M. Zhuang, Q. Liao, and K. Xi, "Integrating Multipolar Structures and Carboxyl Groups in sp^2-Carbon Conjugated Covalent Organic Frameworks for Overall Photocatalytic Hydrogen Peroxide Production," *Angewandte Chemie International Edition* 63 (2024): e202408802.

[S9] W. Chi, J. Wu, Y. Dong, J. Wu, and Y. Zhu, "Accelerated Exciton Dissociation and Charge Transfer via Third-Motif Engineered Conjugated Polymers for Photocatalytic Circulation-Flow Synthesis of H_2O_2," *Angewandte Chemie International Edition* 64 (2025): e202508690.

[S10] C. Wu, Z. Teng, C. Yang, F. Chen, H. B. Yang, L. Wang, H. Xu, B. Liu, G. Zheng, and Q. Han, "Polarization Engineering of Covalent Triazine Frameworks for Highly Efficient Photosynthesis of Hydrogen Peroxide from Molecular Oxygen and Water," *Advanced Materials* 34 (2022): 2110266.

[S11] Z. Gao, F. Liu, Z. Chen, Q. Song, P. J. Cullen, X. Zhang, Z. Zuo, J. Zhong, X. Lu, Z. Hu, R. Liu, Q. Zhang, Y. Yin, and Y. Cai, "Defect-Modulated Oxygen Adsorption and Z-Scheme Charge Transfer for Highly Selective H_2O_2 Photosynthesis in Pure Water," *Nature Communications* 16 (2025): 8889.

[S12] J.-L. Zhou, Y.-F. Mu, M. Qiao, M.-R. Zhang, S.-X. Yuan, M. Zhang, and T.-B. Lu, "Unlocking One-Step Two-Electron Oxygen Reduction via Metalloid Boron-Modified $Zn_3In_2S_6$ for Efficient H_2O_2 Photosynthesis," *Angewandte Chemie International Edition* 64 (2025): e202506963.

[S13] P. Li, F. Ge, Y. Yang, T. Wang, X. Zhang, K. Zhang, and J. Shen, "1D Covalent Organic Frameworks Triggering Highly Efficient Photosynthesis of H_2O_2 via Controllable Modular Design," *Angewandte Chemie International Edition* 63 (2024): e202319885.

[S14] Q. Zhu, L. Shi, Z. Li, G. Li, and X. Xu, "Protonation of an Imine-Linked Covalent Organic Framework for Efficient H_2O_2 Photosynthesis under Visible Light up to 700 nm," *Angewandte Chemie International Edition* 63 (2024): e202408041.

[S15] K. Zhang, L. Tian, J. Yang, F. Wu, L. Wang, H. Tang, and Z.-Q. Liu, “Pauling-Type Adsorption of O_2 Induced by Heteroatom Doped $ZnIn_2S_4$ for Boosted Solar-Driven H_2O_2 Production,” *Angewandte Chemie International Edition* 63 (2024): e202317816.

[S16] S. Yan, Y. Li, X. Yang, X. Jia, J. Xu, and H. Song, “Photocatalytic H_2O_2 Generation Reaction with a Benchmark Rate at Air–Liquid–Solid Joint Interfaces,” *Advanced Materials* 36 (2024): 2307967.

[S17] M. Dai, N. Han, X. Li, J. Shi, H. Chen, Z. Chen, and M. Wang, “Through-Space Charge Transfer Complexes Based on Terpyridine Bi-Functionalization for Efficient Solar-Driven H_2O_2 Production,” *Angewandte Chemie International Edition* 65 (2026): e202521532.

[S18] J. Zhang, H. Lei, Z. Li, F. Jiang, L. Chen, and M. Hong, “Halogen-Modulated 2D Coordination Polymers for Efficient Hydrogen Peroxide Photosynthesis under Air and Pure Water Conditions,” *Angewandte Chemie International Edition* 63 (2024): e202316998.

[S19] A. Chakraborty, A. Alam, U. Pal, A. Sinha, S. Das, T. Saha-Dasgupta, and P. Pachfule, “Enhancing Photocatalytic Hydrogen Peroxide Generation by Tuning Hydrazone Linkage Density in Covalent Organic Frameworks,” *Nature Communications* 16 (2025): 503.

[S20] Z.-Z. Liang, Y. Wang, X.-A. Li, X.-B. Peng, L. Gong, L.-M. Xiao, X. Yang, B. Zou, Y. Zhou, and J.-M. Liu, “Enhanced Piezo-Photocatalytic Water Splitting Activity via Engineering Robust Dipole Moments in Covalent Organic Frameworks,” *Nature Communications* 17 (2026): 729.

[S21] C. Qin, X. Wu, L. Tang, X. Chen, M. Li, Y. Mou, B. Su, S. Wang, C. Feng, J. Liu, X. Yuan, Y. Zhao, and H. Wang, “Dual Donor–Acceptor Covalent Organic Frameworks for Hydrogen Peroxide Photosynthesis,” *Nature Communications* 14 (2023): 5238.

[S22] S. Yang, D. Si, L. Zou, M. Shi, Y. Huang, and R. Cao, “Engineering Photo-Controlled Dynamic Conjugation Switching in Azine-Linked Covalent Organic Frameworks for Boosted Photocatalysis,” *Angewandte Chemie International Edition* 65 (2026): e202518661.

[S23] Y. X. Ye, J. H. Pan, F. Y. Xie, L. Gong, S. M. Huang, Z. F. Ke, F. Zhu, J. Q. Xu, and G. F. Ouyang, “Highly Efficient Photosynthesis of Hydrogen Peroxide in Ambient Conditions,” *Proceedings of the National Academy of Sciences of the United States of America* 118 (2021): e2103964118.

[S24] Q. Liu, H. Bi, R. Zhao, X. Yang, F. Chen, and Z. Shen, “Fully Exposed Silver Clusters Enabling Highly Efficient Photocatalytic H_2O_2 Production in Pure Water,” *Angewandte Chemie International Edition* 64 (2025): e202511687.

[S25] C. Wang, S. Tu, F. Chen, T. Ma, and H. Huang, “Asymmetric Single-Unit-Cell Layer Enriching Polar Inherent Hydroxyls Eliminates Interlayer Electric Field Shielding Effect and In Situ Self-Polarize for Piezocatalytic Water Splitting,” *Advanced Materials* 37 (2025): 2505592.

[S26] B. Feng, Y. Liu, K. Wan, S. Zu, Y. Pei, X. Zhang, M. Qiao, H. Li, and B. Zong, “Tailored Exfoliation of Polymeric Carbon Nitride for Ultra-High Photocatalytic H_2O_2 Evolution,” *Angewandte Chemie International Edition* 63 (2024): e202401884.

[S27] F. Liu, P. Zhou, Y. Hou, H. Tan, Y. Liang, J. Liang, Q. Zhang, S. Guo, M. Tong, and J. Ni, “Covalent Organic Frameworks for Direct Photosynthesis of Hydrogen Peroxide from Water, Air and Sunlight,” *Nature Communications* 14 (2023): 4344.

[S28] D. Shao, L. Zhang, S. Sun, and W. Wang, “Oxygen Reduction Reaction for Generating H_2O_2 through a Piezo-Catalytic Process over Bismuth Oxychloride,” *ChemSusChem* 11 (2018): 527–531.

[S29] J.-N. Lu, J.-J. Liu, L.-Z. Dong, J.-M. Lin, F. Yu, J. Liu, and Y.-Q. Lan, “Synergistic Metal–Nonmetal Active Sites in a Metal–Organic Cage for Efficient Photocatalytic Synthesis of Hydrogen

Peroxide in Pure Water," *Angewandte Chemie International Edition* 62 (2023): e202308505.
[S30] Y. Wei, Y. Zhang, W. Geng, H. Su, and M. Long, "Efficient Bifunctional Piezocatalysis of Au/$BiVO_4$ for Simultaneous Removal of 4-Chlorophenol and Cr(VI) in Water," *Applied Catalysis B: Environmental* 259 (2019): 118084.
[S31] J.-N. Lu, J.-J. Liu, L.-Z. Dong, J.-M. Lin, F. Yu, J. Liu, and Y.-Q. Lan, "Synergistic Metal–Nonmetal Active Sites in a Metal–Organic Cage for Efficient Photocatalytic Synthesis of Hydrogen Peroxide in Pure Water," *Angewandte Chemie International Edition* 62 (2023): e202308505.
[S32] M. Fu, J. Luo, B. Shi, S. Tu, Z. Wang, C. Yu, Z. Ma, X. Chen, and X. Li, "Promoting Piezocatalytic H_2O_2 Production in Pure Water by Loading Metal–Organic Cage-Modified Gold Nanoparticles on Graphitic Carbon Nitride," *Angewandte Chemie International Edition* 63 (2024): e202316346.
[S33] Y.-X. Ye, J. Pan, F. Xie, and G. Ouyang, "Highly Efficient Photosynthesis of Hydrogen Peroxide in Ambient Conditions," *Proceedings of the National Academy of Sciences of the United States of America* 118 (2021): e2103964118.
[S34] J. Feng, Y. Fu, X. Liu, S. Tian, S. Lan, and Y. Xiong, "Significant Improvement and Mechanism of Ultrasonic Inactivation to *Escherichia coli* with Piezoelectric Effect of Hydrothermally Synthesized t-$BaTiO_3$," *ACS Sustainable Chemistry & Engineering* 6 (2018): 6032–6041.
[S35] Z. Xie, X. Chen, W. Wang, X. Ke, X. Zhang, S. Wang, X. Wu, J. C. Yu, and X. Wang, "Variation of Chemical Microenvironment of Pores in Hydrazone-Linked Covalent Organic Frameworks for Photosynthesis of H_2O_2," *Angewandte Chemie International Edition* 63 (2024): e202410179.
[S36] World Health Organization, *Guidelines for Drinking-Water Quality: Fourth Edition Incorporating the First and Second Addenda*, WHO, Geneva, 2022.
[S37] X. Liu, J. L. Yang, J. H. Li, X. L. Li, J. Li, X. Y. Lu, J. Z. Shen, Y. W. Wang, and Z. H. Zhang, "Analysis of Water-Soluble Azo Dyes in Soft Drinks by High Resolution UPLC–MS," *Food Additives & Contaminants: Part A* 28, no. 10 (2011): 1315–1323.
